# Radical‐Mediated In Situ Fluorescence Dye Deposition: A Simple Interfacial Signal Amplification Reaction for Ultrasensitive Immunoassay on Barcode Beads

**DOI:** 10.1002/advs.76670

**Published:** 2026-07-20

**Authors:** Jiayu Zhang, Yao Wang, Xinyi Huang, Haiyu Wu, Qiuning Lin, Hongchen Gu, Li Jiang, Linyong Zhu, Hong Xu

**Affiliations:** ^1^ School of Biomedical Engineering Shanghai Jiao Tong University Shanghai China; ^2^ Optogenetics and Synthetic Biology Interdisciplinary Research Center Shanghai Frontiers Science Center of Optogenetic Techniques for Cell Metabolism School of Pharmacy East China University of Science and Technology Shanghai China

**Keywords:** barcode beads, interfacial reaction, radical‐mediated in situ fluorescence dye deposition (RIFD), signal amplification, suspension chip, ultrasensitive immunoassay

## Abstract

Ultrasensitive detection of low‐abundance protein biomarkers is crucial for early disease diagnosis but remains challenging for the conventional barcode beads‐based suspension chip platform due to the limited detection sensitivity. Here, we report a conceptually novel reaction termed “radical‐mediated in situ fluorescence dye deposition” (RIFD) as a simple interfacial signal amplification strategy to overcome the limitation. This first discovered RIFD follows a universal “three‐element principle”, where the coexistence of beads, free radicals, and dyes suffices for fluorescence labeling, eliminating the pre‐conjugation of dyes to detection probes required in traditional methods. Mechanistic studies reveal that this radical‐triggered RIFD possibly follows three distinct pathways, including amide condensation on lysine residues, covalent biphenyl formation on tyrosine residues, or radical‐dye co‐deposition. It facilitates local and ultrafast (within 5 min) dye‐trapping specifically on target‐positive barcode beads. Consequently, the established RIFD‐based immunoassay achieves a limit of detection of 12 fg/mL for IL‐10, a 100‐fold improvement over the conventional suspension chip method, and also successfully differentiates Alzheimer's disease patients from healthy controls by quantifying low‐abundance plasma p‐Tau217. Multiplexed detection is further validated with a three‐plex cytokines panel. Our reported RIFD represents a powerful in situ fluorescence labeling tool, advancing protein biomarker detection toward the sub‐pg/mL level with broad implications for clinical diagnostics.

## Graphical abstract

A radical‐mediated in situ fluorescence dye deposition (RIFD) reaction containing merely three elements of beads, dyes, and radicals is discovered for the first time, and its underlying mechanism is elucidated. This simple RIFD enables the interfacial and ultrafast deposition of free dyes onto barcode beads, serving as a powerful signal amplification tool for ultrasensitive immunoassay on barcode beads‐based suspension chip platform.

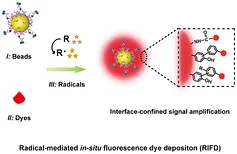

## 1 Introduction

The abnormal expression of proteins is highly relevant to the occurrence and development of diseases; thus, the quantitative detection of protein biomarkers is of great significance for diagnosis, prognosis, and therapeutic evaluation, etc [1, 2, 3]. In particular, the progression of complex diseases, such as cancer and Alzheimer's disease (AD), is often associated with abundance changes of multiple targets [4, 5]. Therefore, high‐throughput multiplexed bio‐detection within a single reaction is a practical and meaningful clinical demand. It not only enhances the diagnostic accuracy and efficiency, but also reduces the excessive consumption of both samples and time [6, 7]. Among the existing systems for multiplexed bioassays, the suspension chip is based on optically encoded barcode beads. It offers quasi‐liquid reaction kinetics, flexible target combination, and high reproducibility, making it one of the most versatile platforms available [8, 9, 10]. Unfortunately, the detection sensitivity of current mainstream suspension chips generally ranges from pg/mL to ng/mL due to the limited number of fluorescence dyes labeled to targets [11, 12]. This sensitivity performance often fails to meet the real clinical requirements for detecting proteins at sub‐pg/mL level, especially in situations where the low‐abundance targets are crucial for early disease diagnosis [13, 14]. Hence, it is a pressing need to overcome only pg/mL detection sensitivity bottleneck on the suspension chip platform to achieve a truly ultrasensitive immunoassay.

To date, in order to upgrade the detection sensitivity of suspension chip‐based immunoassay, two major strategies have been developed. On the one hand, substantial efforts have been made to improve detection performance through various signal amplification methods. Ultrabright nanoparticles are widely used to realize direct fluorescence intensity enhancement of tags per binding event [15, 16, 17]. However, this strategy normally employs large particle sizes to boost fluorescence, which can lead to deteriorated reaction kinetics and low label efficiency [18, 19, 20]. DNA or enzyme‐assisted amplification approaches [21, 22, 23], such as rolling circle amplification (RCA) [24, 25, 26], hybridization chain reaction (HCR) [27, 28], and tyramide signal amplification (TSA) [29, 30, 31], are also utilized to amplify the reporting signals. Nevertheless, DNA‐based methods need strict professional experimental conditions, such as a PCR laboratory, and skilled operators [32, 33]. Meanwhile, enzyme‐triggered amplifications face a high risk of signal diffusion‐induced crosstalk among different barcode beads [34, 35]. Moreover, all these amplification strategies and conventional fluorescence labeling rely on covalent pre‐conjugation of dyes with various partners, such as nucleic acids [33, 36], small molecule compounds [37], and proteins [38, 39]. This process can compromise the performance of dyes or their partners during the complex and time‐consuming coupling steps. On the other hand, as a methodological innovation, digital enzyme‐linked immunosorbent assay (d‐ELISA) has significantly improved detection sensitivity by counting binary signals at the single‐molecule level [40, 41, 42, 43, 44, 45, 46]. Single molecule array (Simoa) is regarded as the pioneer in digital immunoassays that has been successfully commercialized. It utilizes a large number of microwells to isolate targets, ensuring that each microwell contains only a single protein molecule for subsequent enzyme‐catalyzed signal amplification [42, 46]. The absolute counting pattern for targets has remarkably facilitated the achievement of ultra‐sensitivity down to fg/mL level. However, this approach suffers from significant drawbacks, including the complicated manufacturing process of microarray chips, as well as the high cost of the required devices and consumables. These limitations make it difficult to meet the demands of routine clinical practice [34, 47]. Therefore, despite considerable efforts, developing a simple, universal, and ultrasensitive immunoassay with a straightforward and accurate signal generation strategy is still a critical need for the suspension chip platform.

To address these challenges, here we report for the first time a novel interfacial chemical reaction termed “radical‐mediated in situ fluorescence dye deposition” (RIFD) as a powerful signal amplification strategy to achieve an ultrasensitive immunoassay. This serendipitously discovered RIFD reaction follows a simple and universal “three‐element principle”, wherein fluorescence labeling occurs directly on the bead surface when merely beads, free radicals, and dyes coexist in a solution (Figure 1A). Based on this new finding, we established a RIFD‐assisted ultrasensitive immunoassay on a suspension chip platform. As illustrated in Figure 1B, rhodamine B isothiocyanate (RITC) encoded magnetic microbeads (EMMs) with different fluorescence levels were conjugated with capture antibodies to create distinct barcode beads. Upon specific target recognition and enzyme labeling, barcode beads‐based immunocomplexes are formed. Subsequently, enzyme‐triggered free radicals trap a large number of fluorescence dyes originally in solution to rapidly deposit onto corresponding barcode beads. This process leverages the localized interfacial reaction nature of RIFD, providing robust fluorescence amplification without signal crosstalk. Both encoding and reporting signals were conveniently analyzed via flow cytometry (FCM) in a speedy and high‐throughput manner. Compared to the conventional suspension chip immunoassay, our proposed RIFD‐based ultrasensitive method achieves a limit of detection (LOD) of 12 fg/mL for IL‐10, which is approximately a 100‐fold improvement. The clinical utility was validated by analyzing the low‐abundance biomarker p‐Tau217 in human plasma. This allowed differentiation of AD patients from cognitively normal control participants. Furthermore, a three‐plex cytokine assay was also performed as proof of concept to demonstrate the feasibility of multiplexed detection using this interfacial signal amplification approach.

**FIGURE 1:**
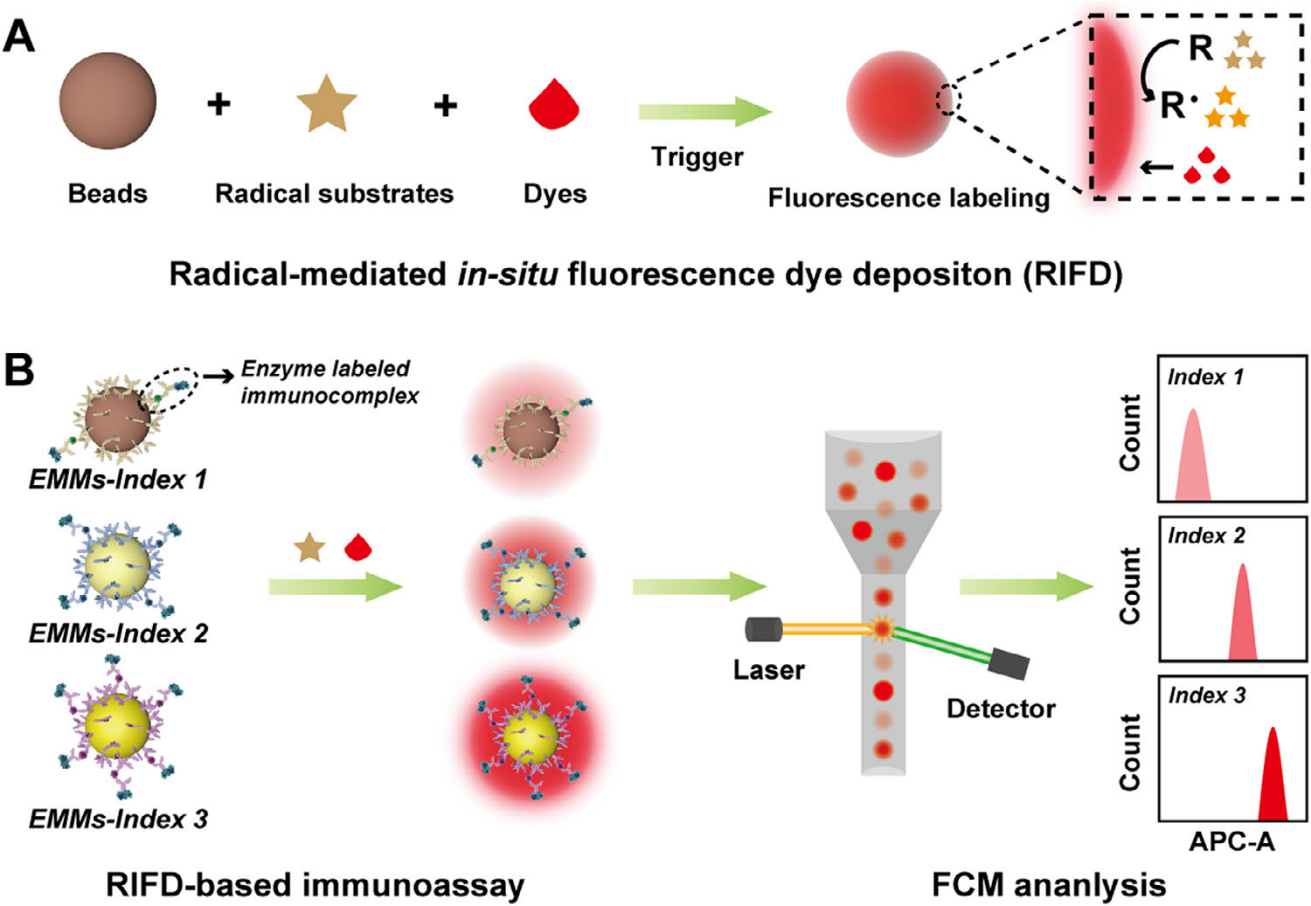
Schematic illustration of (A) the RIFD reaction and (B) its application as an interfacial signal amplification strategy on barcode beads for ultrasensitive immunoassay.

## 2 Results and Discussion

### 2.1 Development of a New RIFD Reaction‐Based Immunoassay

We have been devoted to developing high‐throughput multiplexed and ultrasensitive suspension chip platforms based on innovative micro/nano‐technologies [11, 39, 40, 41, 48]. In the recent investigation, we serendipitously uncovered a previously unreported phenomenon termed RIFD. This interfacial reaction enables a simple and universal fluorescence labeling on the bead surface when merely three elements of beads, free radicals, and dyes coexist in solution. In order to illustrate our new discovery, acetylacetone (ACAC) as the radical substrate and 5‐carboxytetramethylrhodamine (5‐TAMRA) as the fluorescence dye were first mixed with beads in a liquid solution (Figure 2Ai). Free radicals are produced by H_2_O_2_ and horseradish peroxidase (HRP) enzyme. Under these conditions, specific fluorescence signals from dyes are significantly generated on beads, in contrast to the relatively low backgrounds of controls with the absence of HRP (Figure 2Bi–iii). It is intriguing to observe that the fluorescence deposition universally appears for various types of beads, regardless of carboxylation (‐COOH), amination (‐NH_2_), or antibodies‐conjugation (‐Ab). Moreover, this RIFD reaction is suitable for dye 5‐carboxyfluorescein (5‐FAM) as well (Figure 2Biv). Thereafter, another light‐triggered system was attempted to validate the universality of free radicals (Figure 2Aii). Specifically, as a typical photo‐initiator, the substrate of diphenyl(2,4,6‐trimethylbenzoyl)phosphine oxide (TPO) can evolve into radicals under UV light radiation [49]. A distinctly positive signal has been obtained compared to the negative group after all reagents were mixed and triggered by light (Figure 2Bv). These results convincingly demonstrate that RIFD adheres to a simple and universal “three‐element principle”, which can be performed with different beads, dyes and free radicals.

**FIGURE 2:**
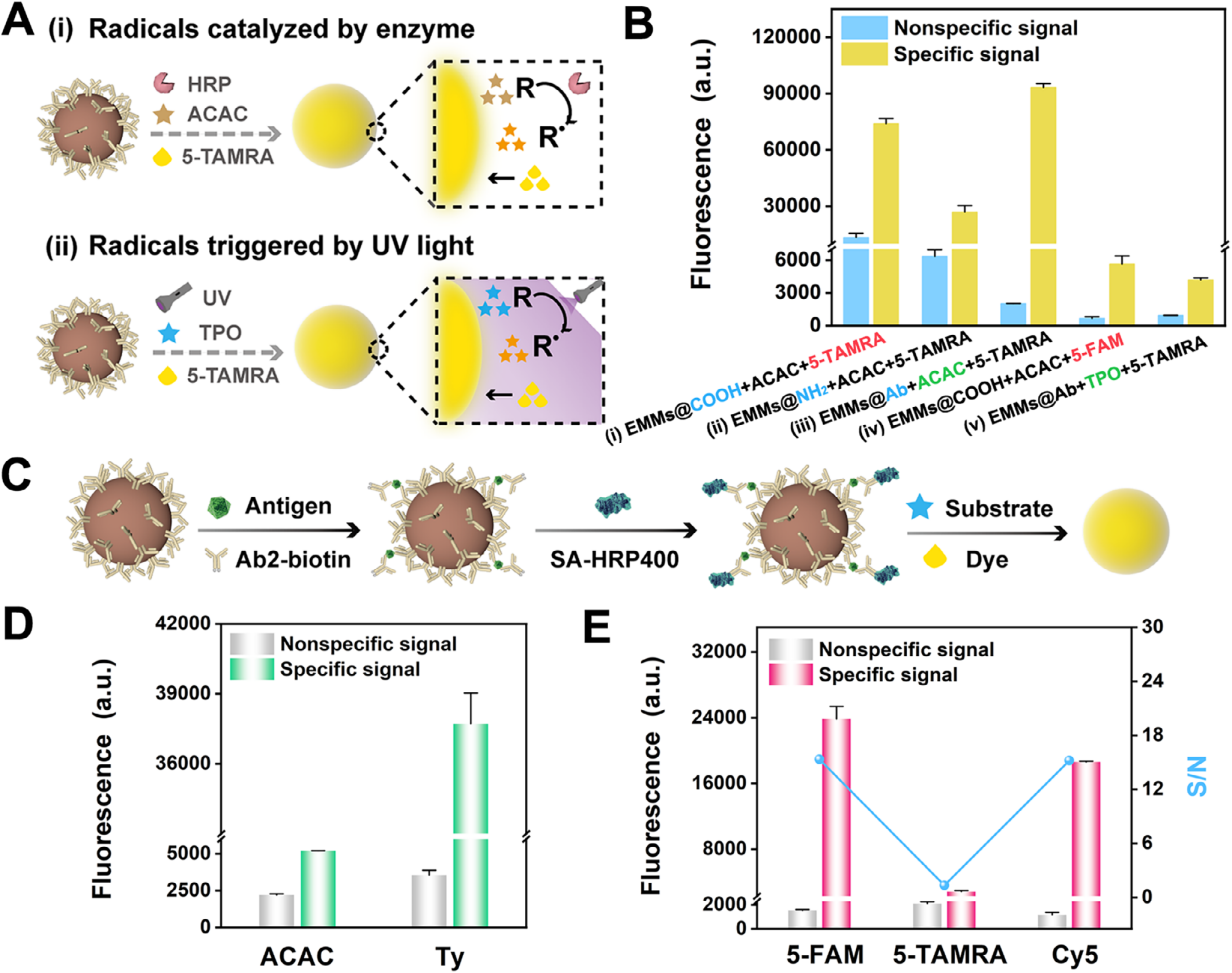
(A) Schematic illustrations of RIFD triggered by (i) enzyme or (ii) UV light in a reagents‐mixed model. (B) FCM analysis of RIFD with various beads, dyes, and radicals. (C) Workflow of RIFD‐based immunoassay. SA‐HRP400: streptavidin‐HRP conjugates with a polymerized number of 400 enzymes. (D) FCM signals for IL‐10 using different radical substrates. (E) Fluorescence intensities and S/N ratios for IL‐10 (6 pg/mL) using different dyes.

Building on this exciting finding, it inspired us to further speculate whether RIFD could utilize the immobilized enzymes on the bead surface via the formation of immunocomplexes to generate in situ reporting signals, thereby constructing a RIFD‐based immunoassay method. Hence, a feasibility verification experiment was designed and performed. Briefly, the cytokine of IL‐10 selected as a model target was mingled with capture antibodies‐conjugated beads (EMMs@IL‐10) and biotinylated detection antibodies (Ab2‐biotin) together to form sandwich immunocomplexes, which were then labeled with streptavidin‐HRP conjugates of SA‐HRP400 (Figure 2C). After adding the radical substrate, H_2_O_2_ and 5‐TAMRA, the enzymes immobilized on the bead surface have successfully triggered the RIFD reaction, as we anticipated. Consequently, clearly positive fluorescence signals appear on beads (Figure 2D). Although both ACAC and tyramide (Ty) are substrates of HRP to produce free radicals in the presence of H_2_O_2_, the radical former of Ty exhibits a more outstanding signal amplification efficiency in RIFD (Figure 2D). Afterward, three typical dyes with different molecule structures and fluorescence wavelengths, including 5‐FAM, 5‐TAMRA, and Cy5 (Figure S1), were chosen to evaluate their performance in RIFD‐based immunoassay. The clear green, yellow, and red fluorescence signals have respectively formed uniform rings on the bead surface (Figure S2). It illustrates that the appearance of free radicals, generated by enzyme‐labeled immunocomplexes, can universally promote the deposition of various dyes. For the performance comparison, sulfo‐cyanine5 (Cy5) and 5‐FAM display remarkably superior signal‐to‐noise (S/N) ratios to 5‐TAMRA (Figure 2E). Finally, Cy5 was selected as the optimal dye for subsequent investigation of RIFD, given its superior S/N ratio and its widespread use as a fluorescence label in the conventional suspension chip platform.

### 2.2 Mechanism Investigation of RIFD Reaction

After the successful establishment of RIFD‐based immunoassay, we next turned to revealing the underlying mechanism of this new‐type reaction. As the antibodies‐modified beads exhibit superior fluorescence deposition to the others (Figure 2B), it leads us to hypothesize that the RIFD reaction occurs more readily between dyes and proteins. Consequently, we simplified the reaction system to include HRP as both the enzyme to produce radicals and the model protein carrier to load dyes. The substrates (Ty or ACAC) and H_2_O_2_ were then added to initiate the RIFD process (Figure S3). By utilizing Cy5 as the dyes, the distinct absorption peaks at 640 nm in the UV–vis absorption spectra are observed for purified protein products compared to the controls (Figure 3A). This indicates the possible covalent‐bond formation between dyes and proteins. Besides, the excitation‐emission fluorescence spectra of positive groups display the same features corresponding to pure Cy5 (Figure 3B), thereby confirming the specific deposition of dyes onto proteins and the intact chemical structure of Cy5 in the presence of free radicals. In terms of fluorescence intensity, a 1.81‐times higher value is recorded under Ty substrate condition than that of ACAC (Figure 3C), which is consistent with the previously observed results in RIFD‐based immunoassay (Figure 2D). The matrix‐assisted laser desorption/ionization time‐of‐flight (MALDI‐TOF) mass spectrometry characterizations show obviously broadening molecular weight distributions of products compared to the original HRP (Figure S4). It suggests that the dyes and proteins undergo a chemical reaction, while the products may not be singular.

**FIGURE 3:**
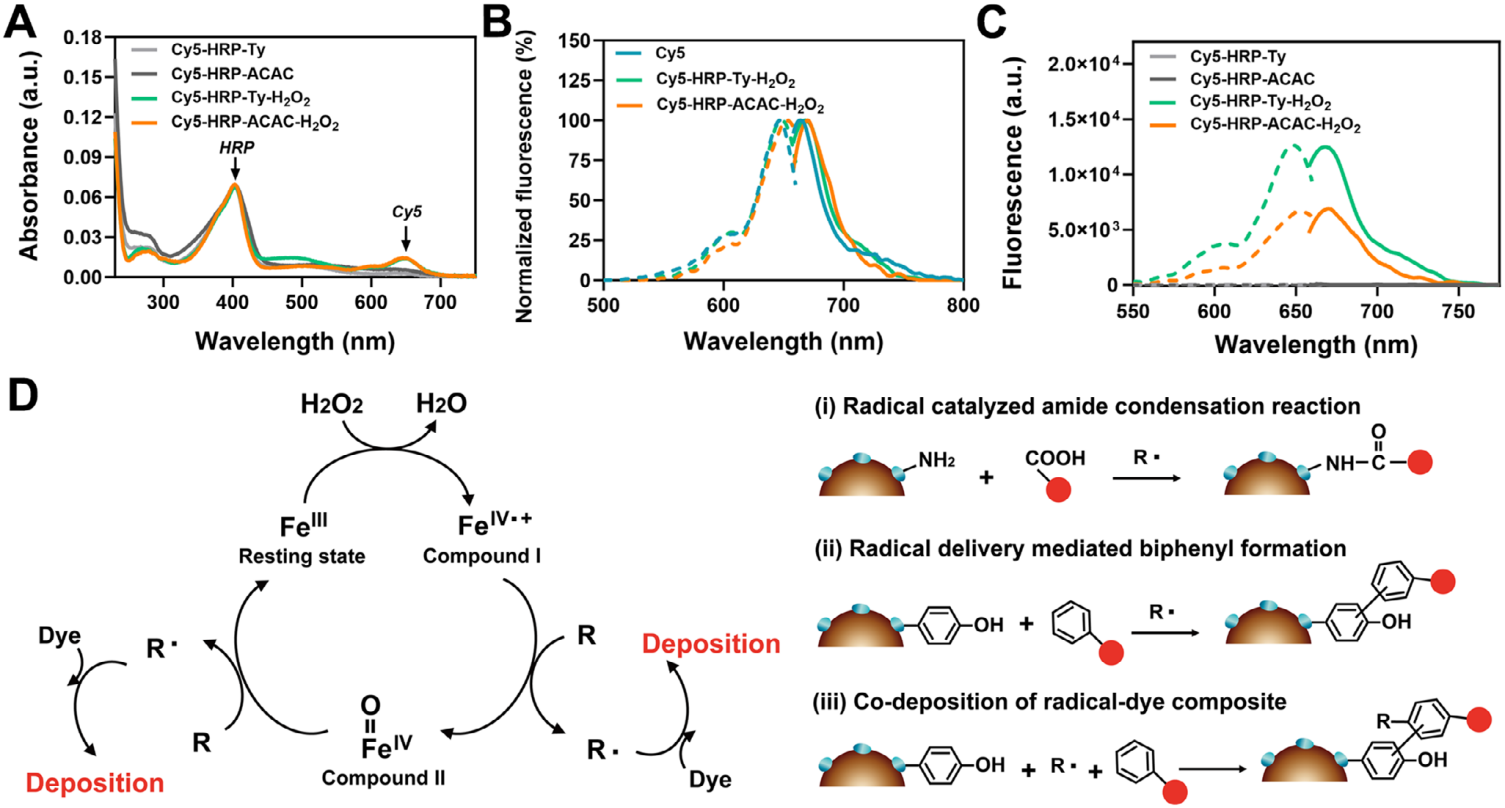
(A) UV–vis absorption spectra of purified protein after RIFD with Cy5 under different conditions. (B) Normalized excitation‐emission fluorescence spectra of the products compared with pure Cy5. (C) Fluorescence spectra of the RIFD products. (D) Proposed RIFD mechanism on bead surface, illustrating three possible deposition pathways.

To further identify the specific sites of dyes covalently linked to proteins, an additional peptide modification resolving was conducted on mass spectrometry (MS) by the enzymatic method [50, 51]. Considering the reactivity of amino acids and the potential reaction mechanism under radical conditions, lysine (K) and tyrosine (Y) were selected for the peptide fragment analysis (Figure S5). When the radical substrate is Ty, it is clear that K at 185 position of HRP has been labeled with an additional molecular weight of 638.21 (Gk(C_33_H_38_N_2_O_7_S_2_ M_W_:638.21)NQCRF) (Figure S6). The exact weight change of Cy5 subtracting a molecule of H_2_O suggests the condensation reaction between ‐COOH from Cy5 and ‐NH_2_ from K. Meanwhile, Y at 245 position is also found to be modified with a molecular weight increase of 654.21 (DNKYy(C_33_H_38_N_2_O_8_S_2_ M_W_:654.21)VNLEEQKGL), indicating that Cy5 is very likely to form a covalent biphenyl structure with Y according to the weight calculation (Figure S7). Actually, this mechanism type is inferred from the deposition pattern of Ty substrate that owns a similar benzene ring structure, as the individual conjugation of Ty on Y was observed as well at positions of 244 and 245 (Ky(C_14_H_17_NO_5_ M_W_:279.11)y(C_14_H_17_NO_5_ M_W_:279.11)VNLEEQKGL) by following the well‐known tyramine deposition approach [31] (Figure S8). As for the ACAC‐mediated Cy5 deposition process, the same rules of both possible carboxyl‐amino condensation on K and covalent biphenyl reaction on Y have been revealed (Figures S9 and S10). Nevertheless, a different product type with a molecular weight of 752.24 (y(C_38_H_44_N_2_O_10_S_2_ M_W_:752.24)VNLEEQKGL) is also formed on Y at 245 position, which is calculated to be the potential co‐deposition of Cy5 and ACAC onto Y (Figure S11). The underlying mechanism may involve the formation of a compound radical between ACAC and the dye molecule, a phenomenon that has been validated in ACAC‐mediated polymerization [52]. To verify whether the mechanisms in RIFD are universal to different dyes, the systematic analysis was performed by replacing Cy5 with 5‐TAMRA. As a result, the spectroscopic experiments demonstrate that 5‐TAMRA can also deposit onto proteins under radical conditions (Figure S12). Furthermore, the MS characterizations suggest that the possible labeling patterns of 5‐TAMRA on proteins are the same as those of Cy5 by using radical substrates of both Ty (Figures S13–S15) and ACAC (Figures S16–S18).

According to the above investigations, a general RIFD reaction mechanism is carefully proposed herein (Figure 3D). The cycle initiates when the heme group of HRP is oxidized by H_2_O_2_, resulting in the formation of water and an intermediate product known as compound I. Subsequently, the reducing substrate (R) donates one electron to compound I and reduces the porphyrin π cation radical, leading to the formation of a radical product (R·) and compound II. In the final step, a second R reduces the Fe (IV) oxoferryl center back to Fe (III), thereby regenerating the initial form of HRP and producing a second R·. All the generated free radicals can further interact with dyes, facilitating their covalent reactions with proteins on the bead surface. It should be objectively clarified that the interfacial proteins are probably not the only reaction sites for RIFD, while they contribute to enhancing deposition efficiency. The deposition mechanisms can be concluded as three patterns, including (i) the radical catalyzed amide condensation reaction, (ii) the radical delivery mediated biphenyl formation, and (iii) the co‐deposition of radical‐dye composite (this depends on substrate type, e.g., ACAC). It is believed that our reported RIFD is a conceptually novel reaction that is totally different from the other well‐known enzymatic amplification strategy of TSA [29, 30, 31]. The detailed comparisons are summarized in Table S1. Additionally, the necessity of free radicals for dye deposition was further attested for a better understanding of the mechanism in RIFD‐based immunoassay. Impressively, the fluorescence signals are obviously generated only when free radicals and dyes are present simultaneously, whereas both the time‐staggered addition of them and the absence of radicals have finally led to failures (Figure S19).

### 2.3 Interfacial Dye‐Trapping Reaction of RIFD‐Based Immunoassay

Free radicals are short‐lived and easily quenched before diffusing far [34, 53], suggesting that RIFD may possess an interfacial reaction feature that enables reporting signal generation directly on target‐positive bead surface. This feature, if realized, would be crucial for preventing crosstalk among different barcode beads. As illustrated in Figure 4A, the verification was conducted by employing two different clusters of beads including negative and positive ones with equal amount. The positive beads were labeled with SA‐HRP400 after the formation of immunocomplexes of IL‐10, then they were mixed with negative ones of blank EMMs@IL‐10 for subsequent RIFD reaction. If our proposed RIFD strategy follows the interfacial dye‐trapping characteristic, Cy5 signals should deposit exclusively on positive beads without spreading to negative ones. This would yield two distinctly separate peaks without interferences as measured via FCM.

**FIGURE 4:**
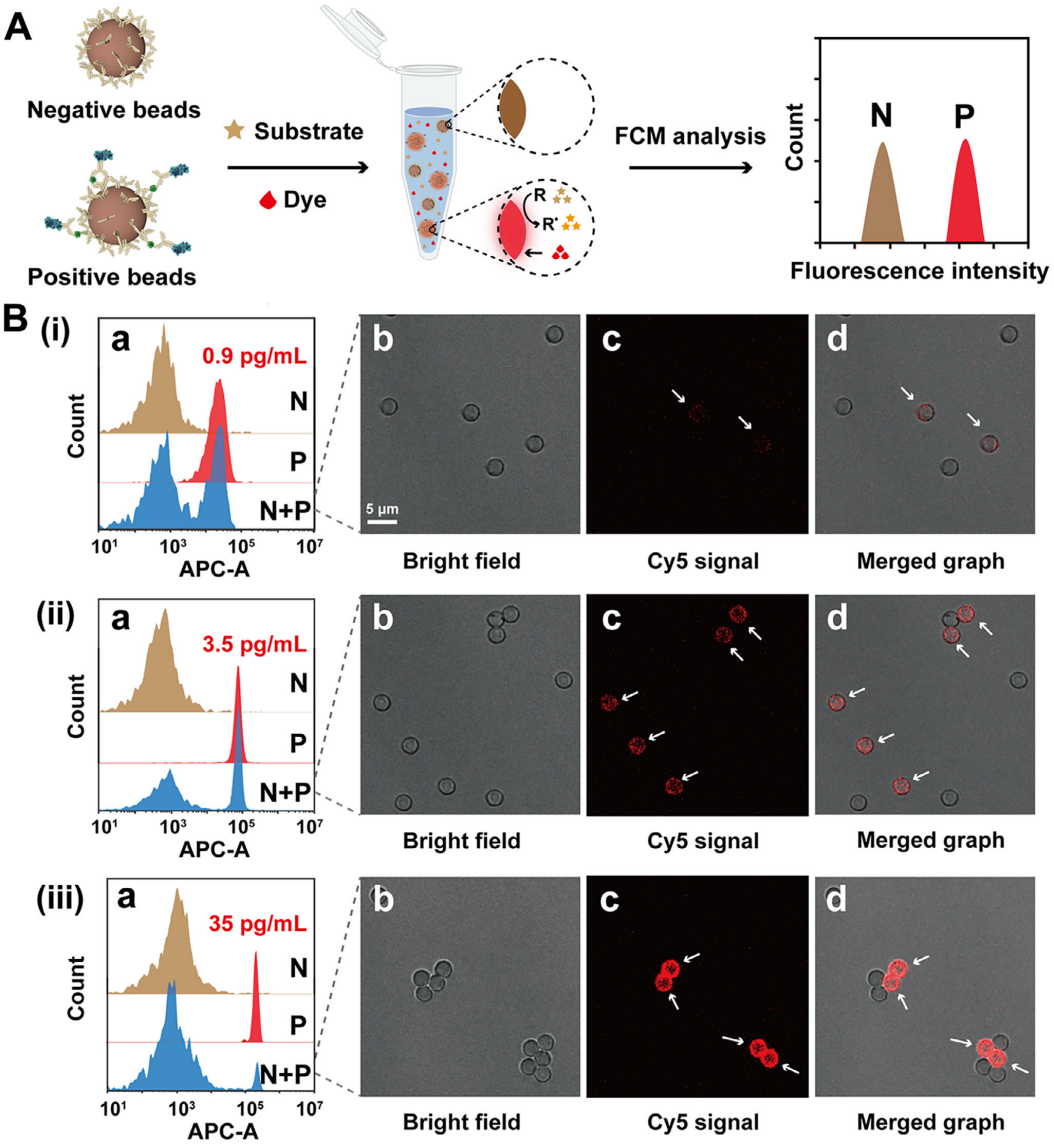
(A) Schematic illustration of interfacial dye‐trapping in RIFD‐based immunoassay using a 1:1 mixture of negative and positive beads. (B) (a) FCM and (b–d) LSCM results for RIFD‐based immunoassay at IL‐10 concentration of (i) 0.9, (ii) 3.5, and (iii) 35 pg/mL. Panels b–d represent (b) bright field, (c) Cy5 fluorescence, and (d) the merged one. The positive beads are marked with white arrows.

To testify the reaction feature of RIFD in a wide dynamic range, three groups containing low (0.9 pg/mL), medium (3.5 pg/mL), and high (35 pg/mL) concentration levels of IL‐10 were used to form different numbers of immunocomplexes on the bead surface (Figure 4Bi–iii). For the analysis of fluorescence intensities via FCM, each group involves three samples: 100% negative beads (designated as N, brown part), 100% positive beads (designated as P, red part), and 50% negative beads mixed with 50% positive beads (designated as N+P, blue part). Compared to the low backgrounds of N, the specific fluorescence signals of P are remarkably strengthened and gradually improve with the concentration of IL‐10 (Figure 4Ba). Notably, the N+P samples always show two clearly discrete peaks, each exactly matching the N and P controls across all target concentration levels. This confirms the in situ deposition of Cy5 on positive beads. In addition, the laser scanning confocal microscope (LSCM) characterizations of N+P samples were also performed (Figure 4Bb–d). Two different types of beads, including non‐fluorescence and red fluorescence (from Cy5, marked with white arrows) in each group are clearly observed while the Cy5 signals increase with IL‐10 concentrations, which is consistent with FCM data. These results have evidently confirmed that RIFD is indeed an interfacial reaction, with in situ dye deposition occurring exclusively on the target‐positive beads. In fact, the average distance between beads is critical for realizing local dye‐trapping in RIFD reaction (Figure S20). This distance‐dependent behavior further underscores the interfacial reaction nature of RIFD, where local dye deposition occurs before diffusion of radicals can cause crosstalk.

### 2.4 Detection Performance Evaluation of RIFD‐Based Immunoassay

In order to achieve the ultrasensitive immunoassay using RIFD reaction as a robust fluorescence signal amplification strategy, several pivotal factors including the polymerization degree of enzymes as well as the concentrations of SA‐HRP400, Cy5, Ty, and H_2_O_2_, were systematically investigated. The corresponding results are discussed in Supporting Information (Section S1.1 and Figure S21A–E). Of note, the RIFD reaction could be completed within 5 min (Figure S21F), demonstrating its rapid and efficient feature. To objectively evaluate the detection performance, IL‐10 was employed as a target and the conventional suspension chip method was simultaneously conducted as a control. Following the formation of immunocomplexes under identical conditions (bead number, antibody concentration, incubation time, and washing step were the same), the label of SA‐HRP400 or SA‐Cy5 was incubated for an equivalent duration of 1 h in the two methodologies. The fluorescence signals can be directly generated on barcode beads for the conventional suspension chip method while the RIFD‐based one needs an additional step of Cy5 deposition (Figure 5A). Nevertheless, the calibration curves clearly show that the fluorescence intensity begins to rise at ∼10^−2^ pg/mL for the RIFD method (Figure 5Bi), which is significantly superior to that of ∼10^0^ pg/mL for the conventional one (Figure 5Ci). Furthermore, both curves display a dynamic range of approximately four orders of magnitude. The fluorescence histograms of FCM at different concentrations demonstrate a consistent conclusion that the signal amplification from RIFD is remarkably powerful compared to the control (Figure 5Bii,Cii). For example, the fluorescence intensity of RIFD‐based immunoassay at 6 pg/mL is 443 times stronger than the background noise, whereas the conventional suspension chip method produces only 3 times improvement with a serious overlap. According to the calibration curves, the LOD defined as the concentration corresponding to the fluorescence intensity of background plus 3 × standard deviation (SD), was carefully calculated. It is determined to be 12 fg/mL for RIFD method (Figure 5Bi) and 1.18 pg/mL for conventional suspension chip (Figure 5Ci), respectively. This nearly 100‐fold sensitivity improvement, which is achieved with a simple RIFD workflow and rapid reaction time, highlights the remarkable efficiency of RIFD as a signal amplification strategy. Compared to SA‐Cy5 conjugated with only a few Cy5 molecules, SA‐HRP400 in RIFD reaction can produce a huge number of free radicals, facilitating sufficient in situ Cy5 deposition on the bead surface for robust signal amplification. This enhancement is particularly beneficial for detecting low‐abundance target, as also confirmed by the LSCM results (Figure S22). Despite the additional step of Cy5 deposition in the RIFD‐based method, the ultra‐short reaction time of merely 5 min in exchange for ultra‐sensitivity is highly cost‐effective. Moreover, the direct use of pure dyes in RIFD for signal generation bypasses the complicated pre‐conjugation of dyes with proteins (e.g., SA or antibodies) required in traditional methods, offering a simpler and more universal approach that avoids possible performance loss.

**FIGURE 5:**
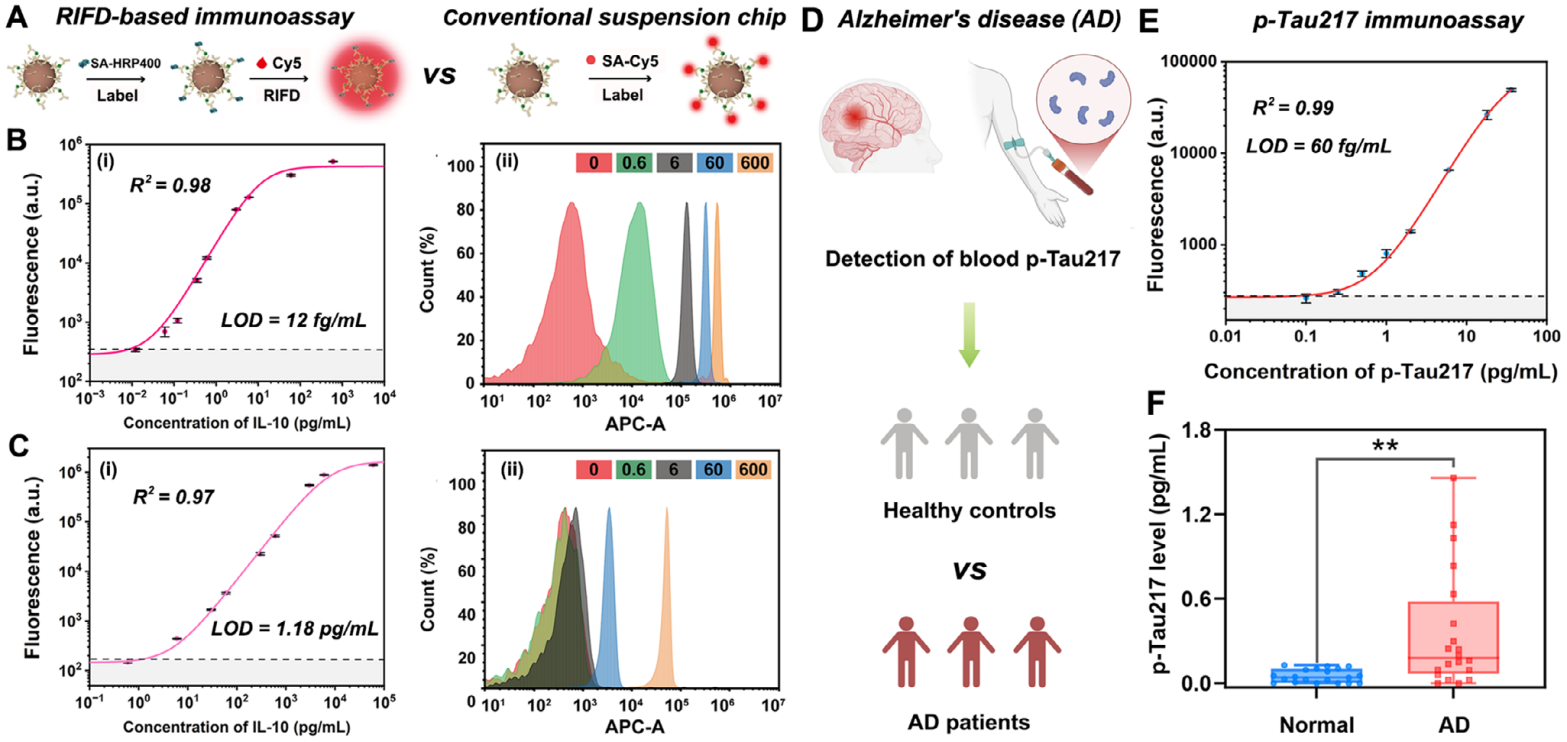
(A) Workflow comparison of RIFD‐based and conventional suspension chip immunoassays. (B, C) (i) Calibration curves and (ii) FCM histograms of (B) the RIFD‐based and (C) conventional assays using IL‐10 at various concentrations (pg/mL). (D) Schematic illustration of p‐Tau217 detection in clinical blood samples. (E) Calibration curve of p‐Tau217 in plasma using RIFD‐based method. (F) Plasma p‐Tau217 levels in cognitively normal controls (n = 20) and AD patients (n = 20). **, *p* < 0.05.

The outstanding performance of our proposed RIFD‐based methodology highlights its potential as a promising tool for fulfilling clinical immunoassay requirements at sub‐pg/mL level. To further validate its practical reliability, we applied this method to detect p‐Tau217, a low‐abundance protein and a core 1 biomarker of AD pathology [54], in clinical blood samples (Figure 5D). In fact, the blood p‐Tau217 detection is a significantly less invasive approach and more suitable for the early diagnosis or screening of AD compared with traditional cerebrospinal fluid (CSF) or PET‐CT analysis [55, 56]. However, the discriminating concentration of p‐Tau217 in blood for early AD patients is typically tens to hundreds of fg/mL, which is much lower than in CSF and thus demands a more reliable ultrasensitive detection method [57]. The optimizations of various reagent concentrations for p‐Tau217 immunoassay were first performed to obtain upgraded performance (Figure S23). The detection results in plasma and buffer display a strong linear correlation with a slope of 0.94, confirming a negligible matrix effect (Figure S24A). Thus, the standard curve for RIFD‐based p‐Tau217 detection in plasma successfully achieves an ultrasensitive LOD of 60 fg/mL (Figure 5E), which is able to satisfy the clinical concentration range. The correlation analysis between RIFD‐based immunoassay and commercial Simoa test for clinical samples of cognitive impairment was also conducted, in which the Spearman coefficient of 0.84 (*p* < 0.05) demonstrates their significant correlations as well as the reliability of our method (Figure S24B). Although the detection sensitivity of the RIFD assay is at a similar level as the commercial Simoa system, the simpler operation and lower cost make it more practical for routine clinical use. We next analyzed plasma samples from 20 clinically diagnosed AD patients and 20 cognitively normal controls utilizing the RIFD‐based assay. Compared to controls, the evidently higher p‐Tau217 levels have appeared for AD patients with a significant difference (p < 0.05) (Figure 5F). These results further underscore the clinical diagnostic potential of our proposed RIFD‐based immunoassay.

### 2.5 Feasibility of RIFD‐Based Multiplexed Immunoassay

For the suspension chip platform, it is highly desirable not only to achieve ultra‐sensitivity but also to maintain the inherent advantage of multiplexed detection. Although the interfacial reaction feature of the RIFD reaction has been well exhibited, to further validate the feasibility of multiplexing, three cytokines of IL‐4, IL‐6, and IL‐10 were selected as model targets for proof‐of‐concept. First, a series of EMMs were constructed by incorporating RITC dyes into beads at blank, low, and high levels according to our previous reports [11, 58]. They were denoted as EMMs@Rb, EMMs@Rl, and EMMs@Rh, respectively. Afterward, they were conjugated with specific capture antibodies of designated indexes to acquire the final barcode beads of EMMs@Rb@IL‐10, EMMs@Rl@IL‐6, and EMMs@Rh@IL‐4 (Figure S25). As depicted in Figure 6A, three distinct fluorescence clusters are clearly resolved when the mixture of barcode beads is decoded using FCM, which is in accordance with the LSCM characterizations showing different brightness (Figure S26). It confirms the successful establishment of a fluorescence‐dependent barcode beads platform.

**FIGURE 6:**
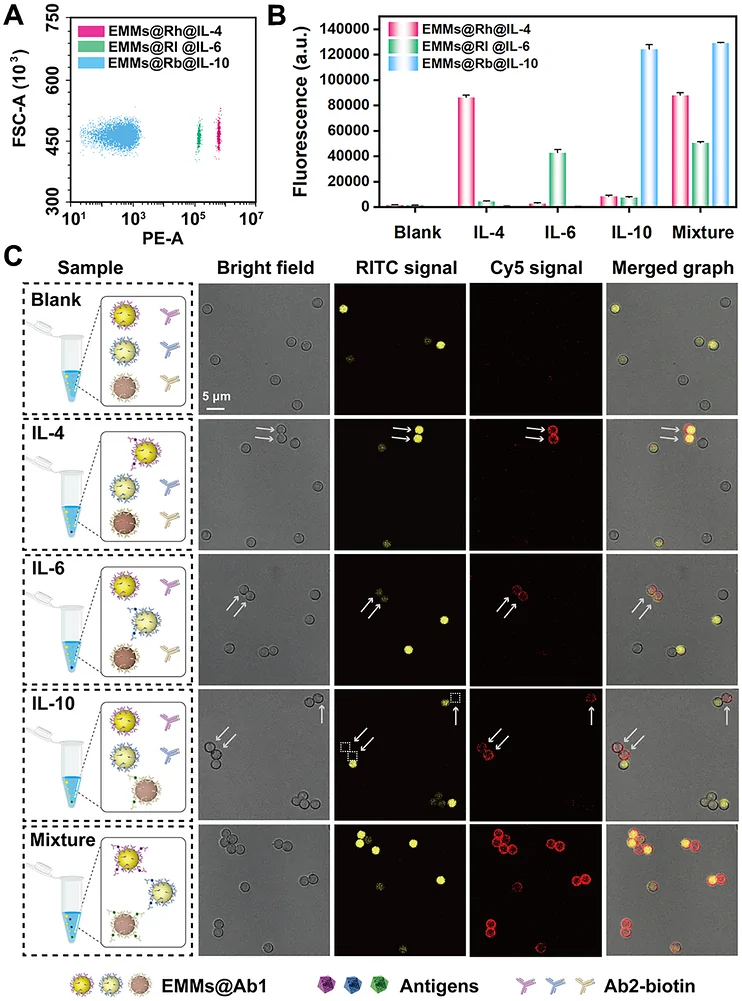
(A) FCM decoding of a mixture of three barcode beads (EMMs@Rh@IL‐4, EMMs@Rl@IL‐6, and EMMs@Rb@IL‐10) in the PE channel. (B) FCM signals of mixed barcode beads after incubation with IL‐4, IL‐6, IL‐10, or a mixture of all three were measured in the APC channel. (C) Schematic and LSCM images of barcode beads under different cytokine conditions. Images show bright field, RITC fluorescence, Cy5 fluorescence, and merged views. The positive barcode beads are marked with white arrows.

To evaluate the specificity of a multiplexed immunoassay based on the RIFD principle, a mixture of barcode beads was challenged with various samples containing no cytokine (blank), each of the three cytokines, or a mixture of all cytokines, respectively. As shown in Figure 6B, the reporting signal of barcode beads specifically elevates only in the presence of its corresponding cytokine, while remaining negligible for the other targets. Ulteriorly, when all three cytokines were added simultaneously, the fluorescence signals of the three barcode beads increased in parallel with comparable intensities to those of each cytokine added individually. This affirms the high specificity of the RIFD‐based multiplexed immunoassay, with an independent fluorescence signal amplification process for each target. Indeed, the short lifetime and limited diffusion distance of radicals inherently prevent crosstalk in RIFD‐based multiplexed immunoassay. Meanwhile, the corresponding schematic illustrations and LSCM images of the mixed barcode beads specific to cytokines under different conditions are also displayed in Figure 6C. It is clearly observed that there is no fluorescence of Cy5 in the blank group, indicating negligible nonspecific adsorption of detection antibodies as well as dyes. Of note, significant Cy5 signals appear on the surfaces of barcode beads in the presence of corresponding cytokines (marked with white arrows). The target index is identified by the RITC fluorescence from barcode beads. For instance, the addition of IL‐4 selectively induces the signal deposition of Cy5 as red rings only on EMMs@Rh@IL‐4 (with the brightest yellow color from RITC) while the other two barcode beads show no response. For the other samples, comparable results are obtained, confirming the high specificity of RIFD‐based multiplexed immunoassay. Importantly, it is the limited diffusion distance of radicals in RIFD that ensures localized interfacial fluorescence deposition and consequently enables independent readout for barcode beads.

## 3 Conclusion

In summary, we report a simple, universal, and rapid RIFD reaction as a robust fluorescence signal amplification strategy for the first time, enabling ultrasensitive immunoassay on barcode beads by utilizing merely three elements of beads, free radicals, and dyes. This newly discovered RIFD reaction, triggered by radicals, proceeds via possible mechanisms including amide condensation, covalent biphenyl formation, or radical‐dye co‐deposition. It enables interfacial and ultrafast (within 5 min) dye‐trapping on target‐positive barcode beads. Our proposed RIFD‐based immunoassay yields a LOD of 12 fg/mL for IL‐10, showing a 100‐fold improvement over the conventional suspension chip method. Its clinical utility is further validated by detecting low‐abundance p‐Tau217 in human plasma to differentiate AD patients from healthy controls. Additionally, a three‐plex cytokine assay demonstrates the feasibility of multiplexed detection with high specificity. We believe this RIFD reaction strategy extends beyond immunoassays as a fundamentally new tool for in situ fluorescence labeling. Its ability to deposit diverse pure dyes onto solid carriers in a rapid, convenient and efficient manner holds potential for broad applications, including chemical biology, molecular biosensing, and interfacial chemistry. For clinical diagnostics, our RIFD‐based signal amplification strategy, serving as a powerful methodology for ultrasensitive immunoassay on barcode beads, holds promise for meeting clinical requirements in the near future.

## 4 Experimental Section

### 4.1 Materials

All reagents acquired from commercially available sources were used as received. *N*‐(3‐dimethylaminopropyl)‐*N’*‐ethylcarbodiimide hydrochloride (EDC), *N*‐hydroxy succinimide (NHS), HRP, 2‐(*N*‐morpholino) ethanesulfonic acid hydrate (MES) were purchased from Sigma‐Aldrich. A series of streptavidin (SA) labeled HRP conjugates including SA‐HRP, SA‐HRP200, and SA‐HRP400 (200 and 400 represent the polymerized number of HRP), were obtained from Fitzgerald Industries International Inc., USA. ACAC, TPO, and dimethylformamide (DMF) were purchased from Sinopharm Chemical Reagent Co., Ltd., China. 2‐((tert‐Butoxycarbonyl)amino)‐3‐(4‐hydroxyphenyl)propanoic acid was bought from Shanghai Haohong Scientific Co., Ltd. Tyramide signal amplification kit was provided by Beijing Biodragon Immunotechnologies Co., Ltd. Different dyes of 5‐FAM, 5‐TAMRA, and Cy5 were obtained from Goyoo Biotech Co., Ltd., China. The pure carboxyl or amino groups functionalized magnetic beads with an average diameter of 3.3 µm were kindly provided by Shanghai So‐Fe Biomedicine Co., Ltd, China. The recombinant antigens of IL‐4, IL‐6, and IL‐10, and the capture as well as biotinylated detection antibodies of IL‐6 were purchased from R&D systems Inc., USA. The capture and biotinylated detection antibodies of IL‐4 and IL‐10 were bought from BioLegend Inc., USA. SA‐Cy5 was provided by Beijing Bioss Biotechnology Co., Ltd., China. All other reagents were obtained from Sinopharm Chemical Reagent Co., Ltd., China. MES buffer containing 0.05 wt% Tween‐20 (MEST) and phosphate buffered saline containing 0.05 wt% Tween‐20 (PBST) were used for the reactions in this study.

### 4.2 Fabrication of Antibody‐Immobilized EMMs

The construction procedure of carboxylated EMMs with a diameter of 3.3 µm was referred to our previously reported works [11, 58]. By adjusting the incorporated dye amount of RITC at blank, low, and high levels, three‐plex barcode beads denoted as EMMs@Rb, EMMs@Rl, and EMMs@Rh could be successfully acquired. Afterward, each kind of EMMs with carboxylic groups were conjugated with specific capture antibodies corresponding to IL‐10, IL‐6, and IL‐4, respectively. Typically, 6.0 × 10^7^ of EMMs were activated in 0.2 mL of MEST (50 mM, pH 6.0) containing EDC (1 mg/mL) and NHS (2 mg/mL) with a rotation for 30 min, and then washed three times with MEST (50 mM, pH 6.0). Subsequently, the activated EMMs were rotationally incubated in 0.2 mL of MEST (50 mM, pH 6.0) containing 0.1 mg of capture antibodies at 37°C for 2 h, and then blocked with PBST (10 mM, pH 7.4) containing 0.5 wt% bovine serum albumin (BSA) and 0.3 wt% glycine at 4°C overnight. Finally, the three antibody‐immobilized barcode beads, which were named as EMMs@Rb@IL‐10, EMMs@Rl@IL‐6, and EMMs@Rh@IL‐4, respectively, were washed three times and stored in 0.1 mL of PBST (10 mM, pH 7.4) containing 0.1 wt% BSA.

### 4.3 RIFD Reaction via Reagents‐Mixed Model

#### 4.3.1 Enzyme‐Induced RIFD Reaction

In a typical reaction, 1 × 10^5^ of beads were dispersed in 0.4 mL of PBS (10 mM, pH 7.4) containing ACAC (30 mM), 5‐TAMRA (1.4 mM), and H_2_O_2_ (0.04 wt%) to form a mixture. Afterward, 0.8 mg of HRP was added for the positive group to induce the RIFD reaction and the whole system was rotationally incubated at 25°C for 30 min. As a control, the absence of HRP was set for negative group to avoid the occurrence of RIFD. Finally, the sample was washed with PBS (10 mM, pH 7.4, 0.1 wt% SDS) and then resuspended in water for analysis on FCM.

#### 4.3.2 Light‐Triggered RIFD Reaction

As for the light‐triggered system, 1 × 10^5^ of beads were suspended in 0.2 mL of DMF containing TPO (2 mM) and 5‐TAMRA (1.4 mM). With the UV light radiation of 20 s, the RIFD reaction was initiated and the mixture was further rotationally conducted at 25°C for 30 min. Finally, the sample was washed with PBS (10 mM, pH 7.4, 0.1 wt% SDS) and then redispersed in water for further FCM analysis.

### 4.4 Investigation of RIFD Reaction Mechanism

The simplified RIFD reaction for mechanism study was performed by incubating HRP (8 mg) with dyes (Cy5 or 5‐TAMRA, 1.4 mM) in 4 mL of PBS. Then, the above mixture was divided into four aliquots, each subjected to one of the following conditions and shaken at 25°C for 30 min: (1) addition of Ty substrate (10 mM) and H_2_O_2_, (2) addition of Ty (10 mM) only as a control, (3) addition of ACAC substrate (10 mM) and H_2_O_2_, (4) addition of ACAC (10 mM) only as a control. After the reaction, the products were purified using a 10 kDa molecular weight cut‐off ultra‐filtration centrifuge tube. The solutions were centrifuged and the products were washed with PBS repeatedly until the filtrate showed no detectable absorbance from free dyes, indicating the complete removal of unreacted molecules.

Afterward, the purified proteins were incubated with 0.5 µL of dithiothreitol (DTT, 1 M) at 37°C for 30 min. Subsequently, 2 µL of iodoacetamide (1 M) was added and the mixture was kept in the dark for 20 min. The protein solution was then transferred to a 10 kDa MWCO ultrafiltration device (Sartorius, PN: VN01H02). Except for using a 5 µg/mL chymotrypsin solution for the digestion step, the subsequent sample preparation followed the standard Filter‐Aided Sample Preparation (FASP) protocol [50, 51]. The collected enzymatic digest was desalted using a micro‐desalting column. The samples were analyzed using a nano‐liquid chromatography coupled to a high‐resolution hybrid mass spectrometer (LC‐MS). Peptides were separated on a house‐packed C18 reversed‐phase analytical column (75 µm × 20 cm, 1.9 µm). Mobile phase A consisted of 99.9% water and 0.1% formic acid, and mobile phase B consisted of 80% acetonitrile and 0.1% formic acid. The mass spectrometer (MS) was operated in positive electrospray ionization (ESI+) mode with a spray voltage of 1700 V. Raw data files were processed using PEAKS Studio (version 12.5). MS/MS spectra were searched against a database constructed from the target protein sequences. The new modifications on K and Y were set as variable modifications. Carbamidomethyl on cysteine (+57.02 Da) was searched as a fixed modification. The mass tolerance of precursor and fragment ions was set at 10 ppm and 0.02 Da, respectively. A non‐specific enzymatic cleavage search was performed. Proteins and peptides were identified with a‐10lgP score threshold ≥ 20. All the modified peptides were manually validated.

### 4.5 RIFD‐Based Immunoassay on Suspension Chip

As a proof of concept, the cytokine detection of IL‐10 was first conducted to explore the performance of our proposed RIFD‐based methodology. In a typical assay, 1 × 10^4^ of EMMs@Rb@IL‐10 were mixed with 0.25 µg of biotinylated detection antibodies and testing sample of IL‐10 with various concentrations in a 0.2 mL of PBST (10 mM, pH 7.4) reaction system. After an incubation at 37°C for 1 h to form immunocomplexes on barcode beads, the sample was magnetically separated and washed with PBST (10 mM, pH 7.4). Then, SA‐HRP400 with a concentration of 46.9 pM in 40 µL of PBST (10 mM, pH 7.4, 1 wt% casein) was added to the sample and incubated for another 1 h at 37°C. Thereafter, the sample was washed by magnetic separation and fed with a mixture of Ty substrate (50 µg/mL), H_2_O_2_ (0.02 wt%), and Cy5 (27.5 µM) in 0.8 mL of PBS (10 mM, pH 7.4) for a further signal amplification process at 25°C. Finally, the sample was washed with PBS (10 mM, pH 7.4, 0.1 wt% SDS) and resuspended in water for FCM analysis. Furthermore, a three‐plex detection of IL‐10, IL‐6, and IL‐4 was also implemented to verify the applicability of the RIFD method for multiplexed immunoassay. Herein, 1 × 10^4^ of each kind barcode beads (EMMs@Rb@IL‐10, EMMs@Rl@IL‐6 and EMMs@Rh@IL‐4) as well as the corresponding three biotinylated detection antibodies were mixed with samples containing different cytokines, while all the other procedures were adopted as the aforementioned ones with optimal parameters.

### 4.6 Conventional Suspension Chip Immunoassay

A conventional suspension chip immunoassay for the detection of IL‐10 was performed as a control group to compare with the RIFD‐based method we established in this work. The first step was the formation of sandwich‐structured immunocomplexes on barcode beads, which was totally the same as that described in Section 4.5. Afterward, 4 µL of SA‐Cy5 (0.01 mg/mL) was added to the sample and incubated in 0.2 mL of PBST (10 mM, pH 7.4) for another 1 h at 37°C, followed by washing with PBS (10 mM, pH 7.4, 0.1 wt% SDS). Finally, the sample was resuspended in water and analyzed by FCM.

### 4.7 Immunoassay of p‐Tau217 for Clinical Plasma Samples

First, the capture antibodies of p‐Tau217 were conjugated on barcode beads (denoted as EMMs@Rb@p‐Tau217) by following the procedure in Section 4.2. To establish the RIFD‐based p‐Tau217 immunoassay for clinical applications, 1 × 10^4^ of EMMs@Rb@p‐Tau217 were mixed with 45 µL of a human plasma sample of p‐Tau217 in 155 µL of PBST (10 mM, pH 7.4) that contains 0.62 µg of biotinylated detection antibodies. After the formation of immunocomplexes with an incubation at 37°C for 1 h, all the protocols including enzyme label and RIFD‐based signal amplification reaction were adopted as the descriptions in Section 4.5 with optimal parameters. The plasma samples of clinically diagnosed AD patients (n = 20) and health controls (n = 20) were collected from Shanghai Fifth People's Hospital, with approval from its ethics committee (approval ID: 2024‐005×1).

### 4.8 Characterization

Both particle number counting and FCM analyses of EMMs were carried out on a NovoCyte 2040R device (ACEA, USA). The FITC (530 ± 15 nm), PE (572 ± 14 nm), and APC (675 ± 15 nm) channels were used for obtaining detection signals from dyes of 5‐FAM, 5‐TAMRA, and Cy5 with excitation lasers of 488 nm, 561 nm, and 640 nm, respectively. For multiplexed assays, the channels of PE (572 ± 14 nm) and APC (675 ± 15 nm) were utilized for decoding of RITC‐coded EMMs and reading detection signals from Cy5, respectively. LSCM images were recorded on a TCS SP5 system (Leica, Germany), where channel 1 (508‐550 nm, for 5‐FAM signal), channel 2 (581‐600 nm, for 5‐TAMRA or RITC signal) and channel 3 (653‐700 nm, for Cy5 signal) were chosen as detectors with excitation sources of 488 nm, 561 nm and 633 nm, respectively. The UV–vis absorption and fluorescence spectra of the purified protein were measured via a UV–vis spectrometer (Agilent Cary 3500, USA) and a fluorescence spectrophotometer (PerkinElmer FL 6500, USA), respectively. MALDI‐TOF mass spectrometry characterizations were conducted on a MALDI‐7090 instrument (Shimazu, Japan). The liquid chromatography coupled mass spectrometer (LC‐MS) analysis was performed on a Vanquish Neo UHPLC‐Orbitrap Exploris 480 system (Thermo Fisher Scientific, USA).

## Funding

This study was supported by the National Key Research and Development Program of China (Grant nos. 2023YFB3210301, 2024YFA0917700, 2024YFA1803501 and 2023YFA1802000), the National Natural Science Foundation of China (Grant nos. 82272122, 82172062, and 22507071), the SJTU Trans‐Med Awards Research (Grant no. 20250202), the Fundamental Research Funds for the Central Universities (Grant nos. YG2024ZD12 and YG2024QNA09), and the Natural Science Foundation of Shanghai (Grant no. 24ZR1432500).

## Conflicts of Interest

The authors declare no conflicts of interest.

## Supporting information




**Supporting File**: advs76670‐sup‐0001‐SuppMat.docx.

## Data Availability

The data that supports the findings of this study are available in the supplementary material of this article.
