# Supplementary material for: Radical‐Mediated In Situ Fluorescence Dye Deposition: A Simple Interfacial Signal Amplification Reaction for Ultrasensitive Immunoassay on Barcode Beads
Source: Adv Sci (Weinh). 2026 Jul 20:e76670. Online ahead of print. doi: 10.1002/advs.76670 (PMC13383694; doi:10.1002/advs.76670)
Supplement: Supplementary file 1 — Supporting File: advs76670‐sup‐0001‐SuppMat.docx. [file ADVS-9999-e76670-s001.docx]

Supporting Information

Radical-Mediated In-Situ Fluorescence Dye Deposition: A Simple Interfacial Signal Amplification Reaction for Ultrasensitive Immunoassay on Barcode Beads

Jiayu Zhang^1^, Yao Wang^1,^*, Xinyi Huang^1^, Haiyu Wu^1^, Qiuning Lin^1^, Hongchen Gu^1^, Li Jiang^1,2,^*, Linyong Zhu^1^, and Hong Xu^1,^*

1. School of Biomedical Engineering, Shanghai Jiao Tong University, Shanghai, China

Li Jiang

2. Optogenetics & Synthetic Biology Interdisciplinary Research Center, Shanghai Frontiers Science Center of Optogenetic Techniques for Cell Metabolism, School of Pharmacy, East China University of Science and Technology, Shanghai, China

E-mail: [wangyao@sjtu.edu.cn](mailto:wangyao@sjtu.edu.cn) (Yao Wang), jiang.li@ecust.edu.cn (Li Jiang), [xuhong@sjtu.edu.cn](mailto:xuhong@sjtu.edu.cn) (Hong Xu)

**S1. Results and Discussion**

**S1.1. Enhancement of Signal Amplification Efficiency**

Herein, fluorescence intensities acquired via FCM without or with IL-10 addition are defined as nonspecific or specific signals, and the ratio of S/N is utilized to evaluate the performance. As a catalytic unit to produce free radicals, the polymerization degree of enzymes plays a crucial role in signal amplifications. It is predicable that highly polymerized enzymes can provide puissant catalytic ability but may also cause a low labeling efficiency due to poor reaction kinetics. To balance these two aspects, a series of polymeric HRP enzymes including SA-HRP, SA-HRP200 and SA-HRP400 were selected for assessment. It is obvious that SA-HRP400 owns the strongest signal amplification ability (**Figure S21A**) and the labeling efficiency gradually increases with the concentration of SA-HRP400 (**Figure S21B**). Considering reagent consumption, a concentration of 46.9 pM is ultimately adopted even though the best S/N plateau has not yet been reached. Afterwards, the concentrations of critical components relating to Cy5, Ty and H_2_O_2_, which affect the dye deposition in RIFD reaction, were further examined. As exhibited in **Figure S21C**, the specific fluorescence intensity progressively elevates at first and then shifts to a declining trend as the Cy5 concentration rises, which is ascribed to the self-quenching effect of dyes with an excessive packing density on bead surface.^[S1, S2]^ Meanwhile, the background noise grows monotonically, leading to an optimal S/N value at a Cy5 concentration of 27.5 μM. As we know, the background signal originates from nonspecific adsorptions of both dye itself and detection antibody or enzyme. The background comparison of mode 4 (81±29, signal only from dye) with mode 1 (235±11, total background signal) in **Figure S19** demonstrates that the nonspecific signal is mainly caused by the adsorption of detection antibody or enzyme which could trigger subsequent signal amplification. Moreover, a similar tendency appears in the regulation of Ty concentration as well (**Figure S21D**). With an increasing Ty amount, the positive signal remarkably improves as more generated free radicals facilitate Cy5 loading on barcode beads. However, the fluorescence intensity dramatically drops when the concentration becomes too high, which is reasonable because excessive Ty radicals tend to form self-quenching dimers^[S3-S5]^ rather than mediating Cy5 deposition on barcode beads. It is definite that both specific signal and S/N value have reached their maximum at a Ty concentration of 50 μg/mL. As for the supplied amount of H_2_O_2_, a clear side effect on signal amplification is observed when more H_2_O_2_ is employed, which is attributed to the peroxidase inactivation.^[S4, S6]^ As a result, a concentration of 0.02 wt% H_2_O_2_ is deemed appropriate (**Figure S21E**). Finally, the parameter of reaction time for RIFD was confirmed. It is worth mentioning that a significant specific signal is well generated within 5 min and remains stable thereafter, demonstrating that this reaction is extremely rapid and efficient (**Figure S21F**).

**S2. Supplementary Figures and Tables**

**
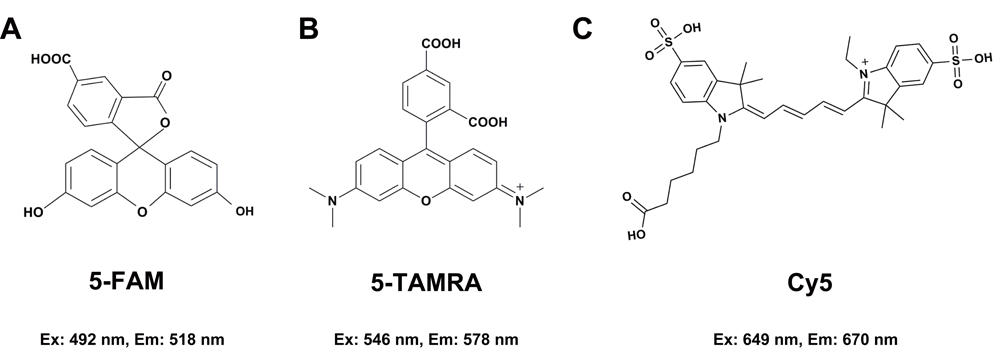
**

**Figure S1.** Molecule structures and fluorescence wavelengths of different dyes including (A) 5-FAM, (B) 5-TAMRA and (C) Cy5.

**
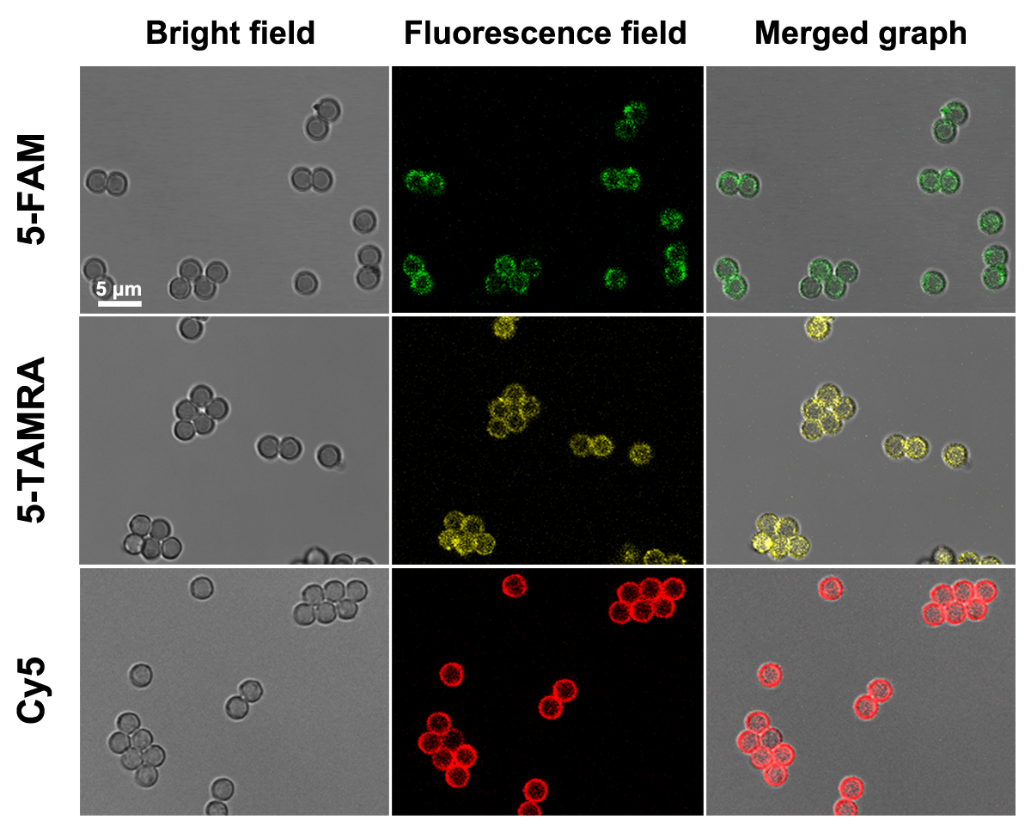
**

**Figure S2.** LSCM images of beads after IL-10 immunoassay using different fluorescence dyes. Columns from left to right: bright field, fluorescence, and merged images.


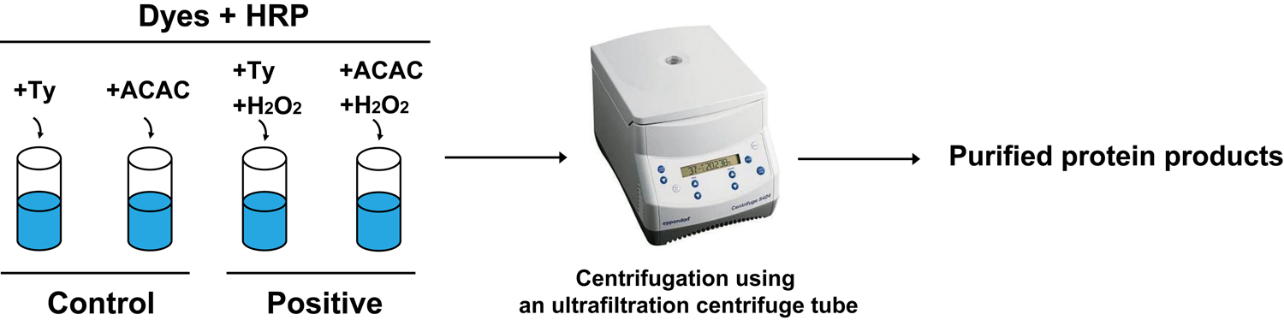


**Figure S3.** Illustration of the simplified RIFD process for reaction mechanism investigation.


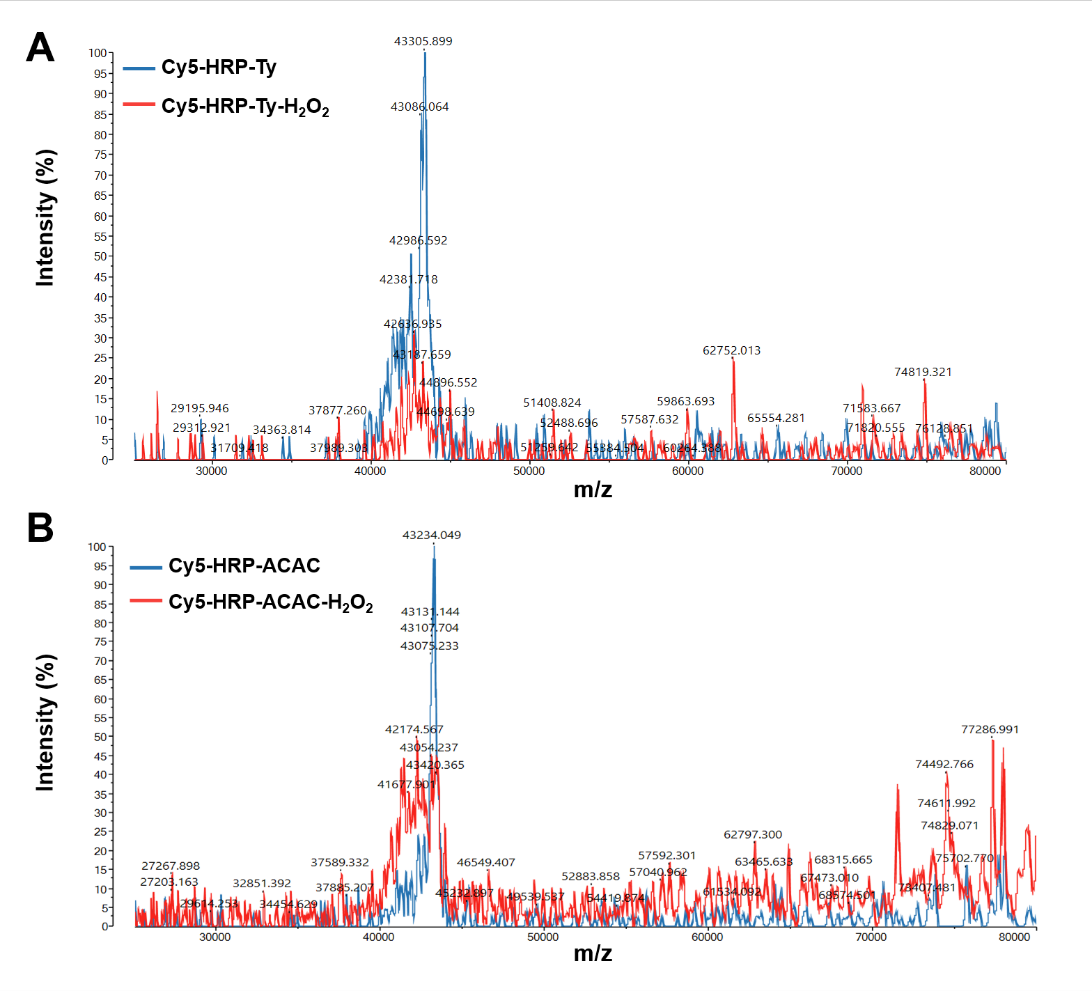


**Figure S4.** MALDI-TOF mass spectrometry analysis of the purified HRP products after RIFD reaction with Cy5 utilizing different radical substrates: (A) Ty, (B) ACAC.

**
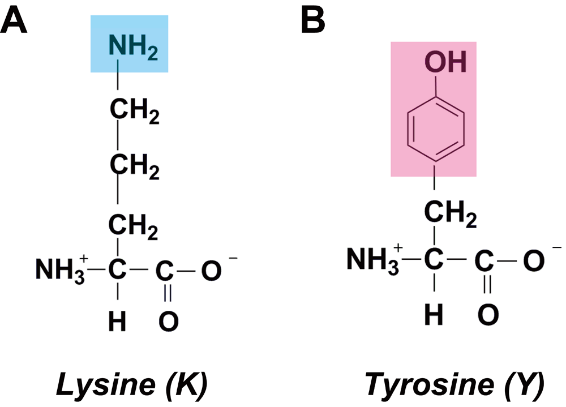
**

**Figure S5.** Molecule structures of K and Y, and these two amino acids were selected for the peptide fragment analysis.

**
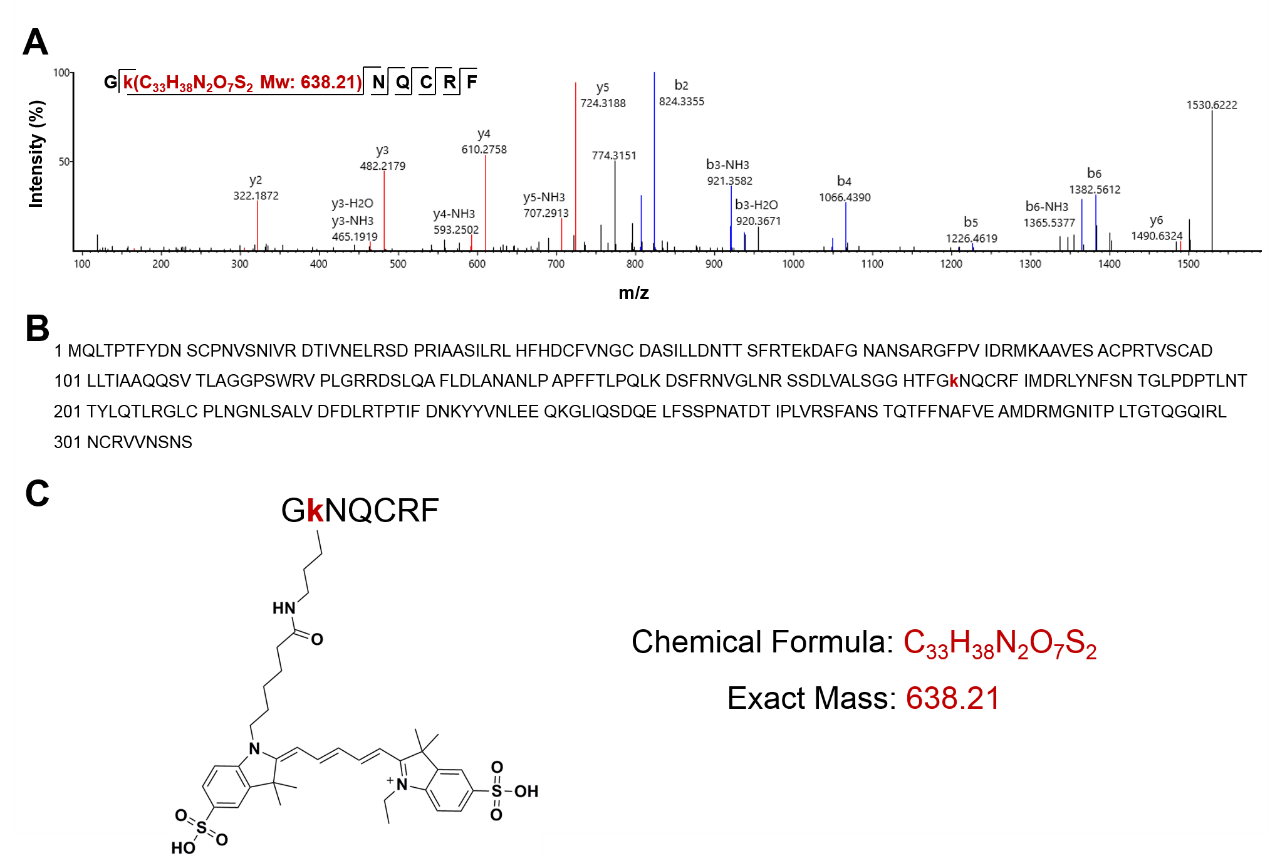
**

**Figure S6.** The peptide modification resolving of the purified HRP products after RIFD reaction with Cy5 and Ty. (A) MS data analysis. (B) The amino acid sequence of HRP and the dye labeled position. (C) The situation of Cy5 labeled on K.

**
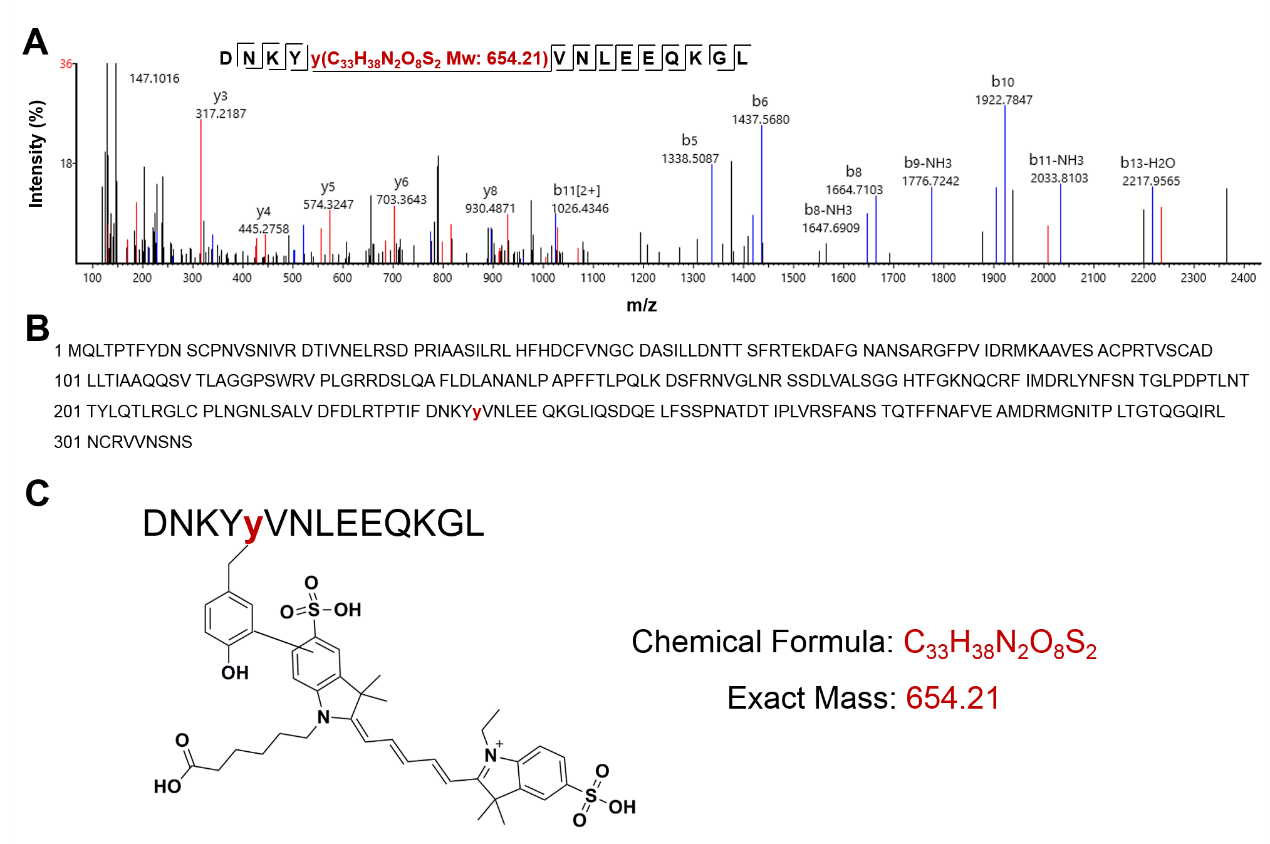
**

**Figure S7.** The peptide modification resolving of the purified HRP products after RIFD reaction with Cy5 and Ty. (A) MS data analysis. (B) The amino acid sequence of HRP and the dye labeled position. (C) The situation of Cy5 labeled on Y.

**
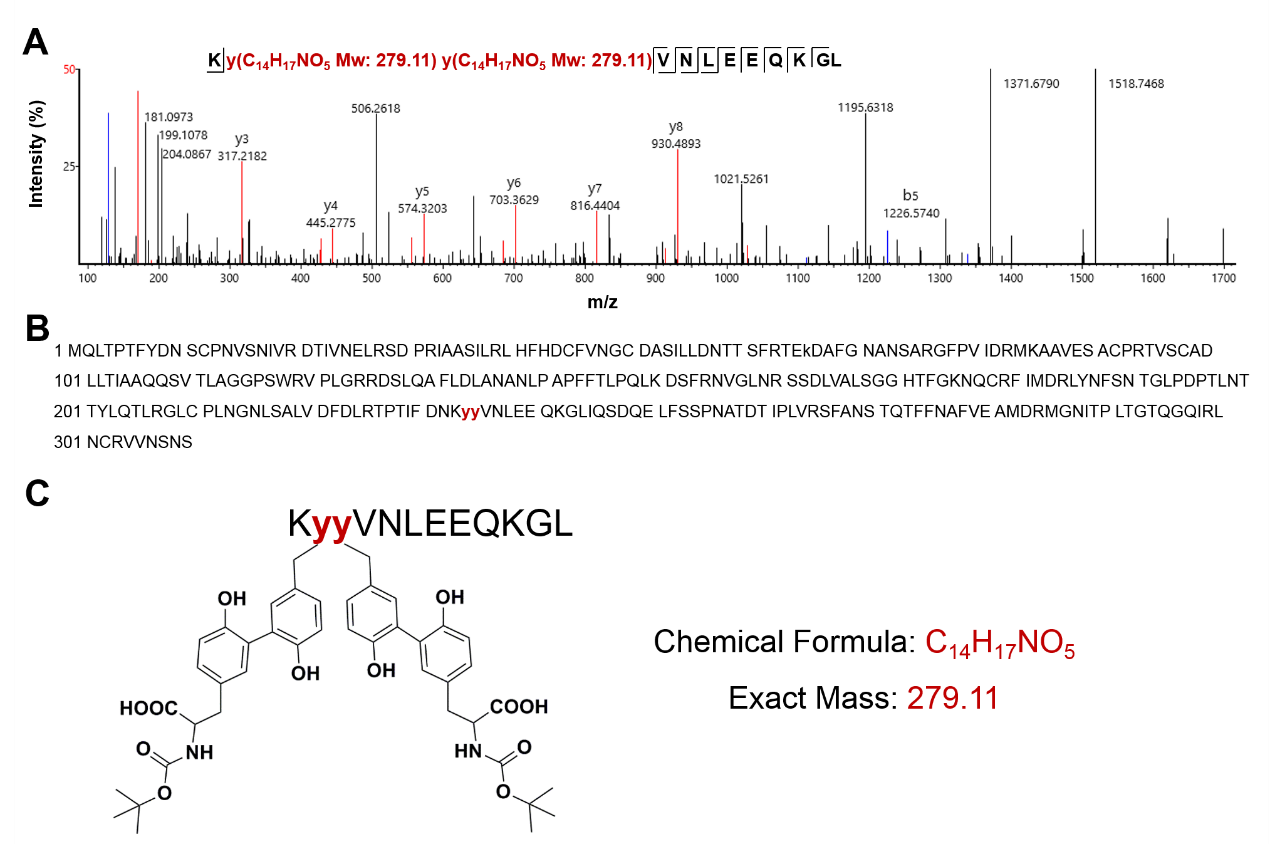
**

**Figure S8.** The peptide modification resolving of the purified HRP products after RIFD reaction with Cy5 and Ty. (A) MS data analysis. (B) The amino acid sequence of HRP and the Ty deposited position. (C) The situation of Ty deposited on Y.

**
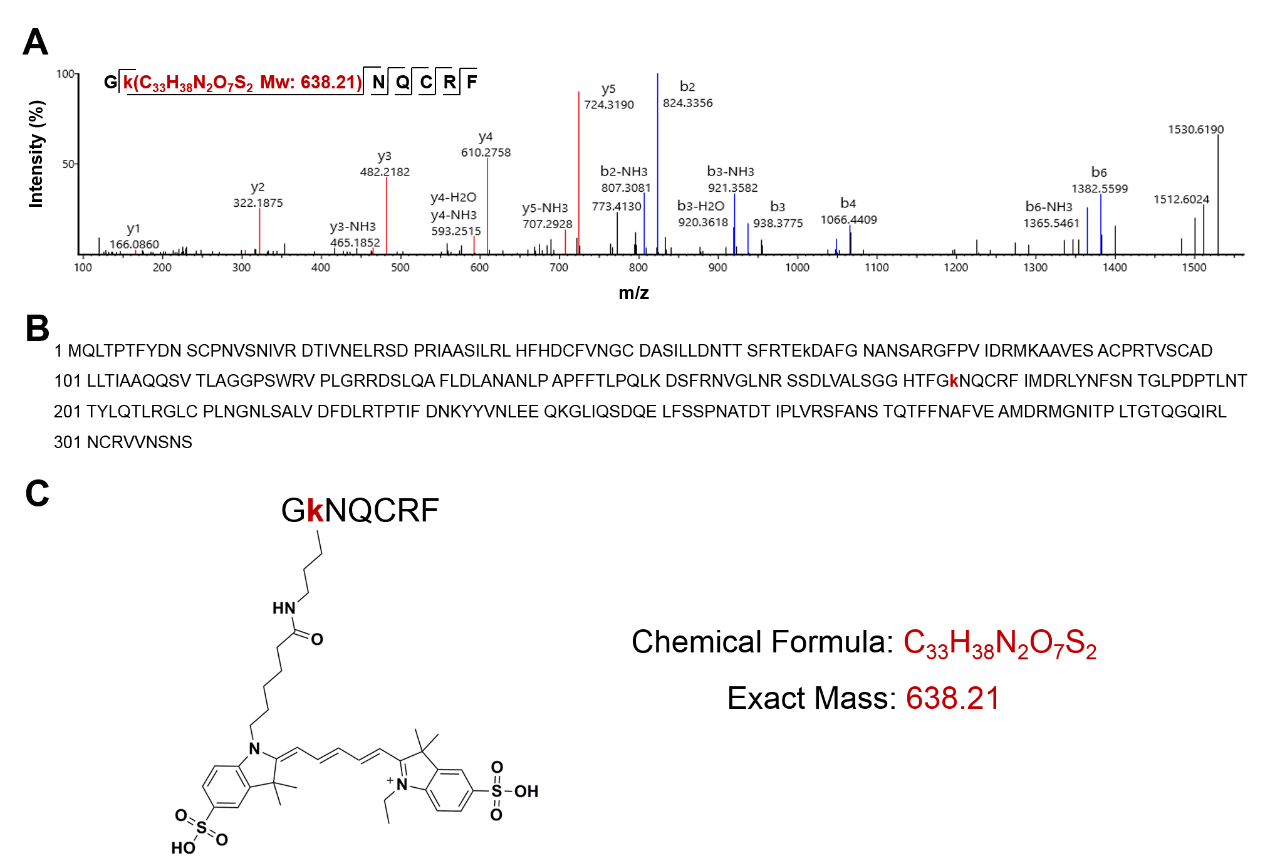
**

**Figure S9.** The peptide modification resolving of the purified HRP products after RIFD reaction with Cy5 and ACAC. (A) MS data analysis. (B) The amino acid sequence of HRP and the dye labeled position. (C) The situation of Cy5 labeled on K.

**
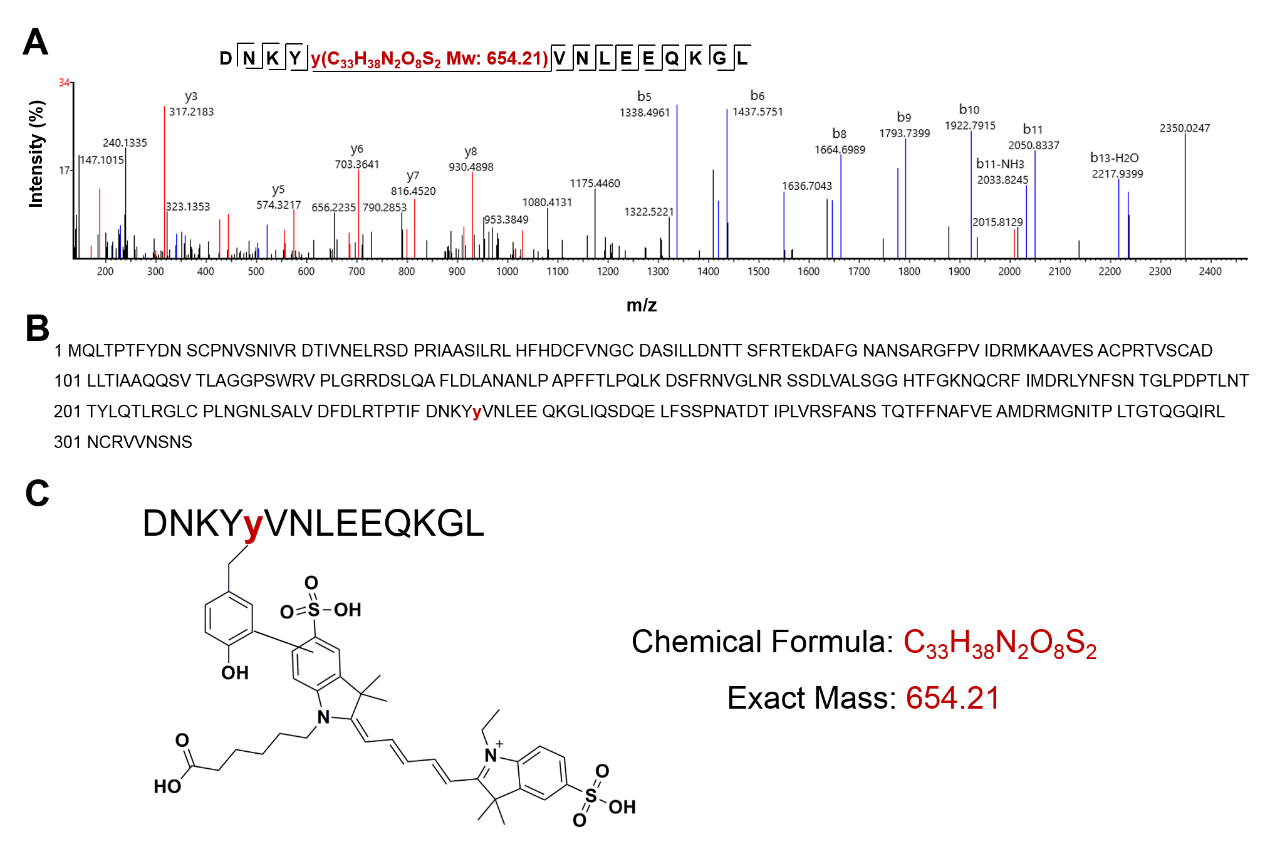
**

**Figure S10.** The peptide modification resolving of the purified HRP products after RIFD reaction with Cy5 and ACAC. (A) MS data analysis. (B) The amino acid sequence of HRP and the dye labeled position. (C) The situation of Cy5 labeled on Y.

**
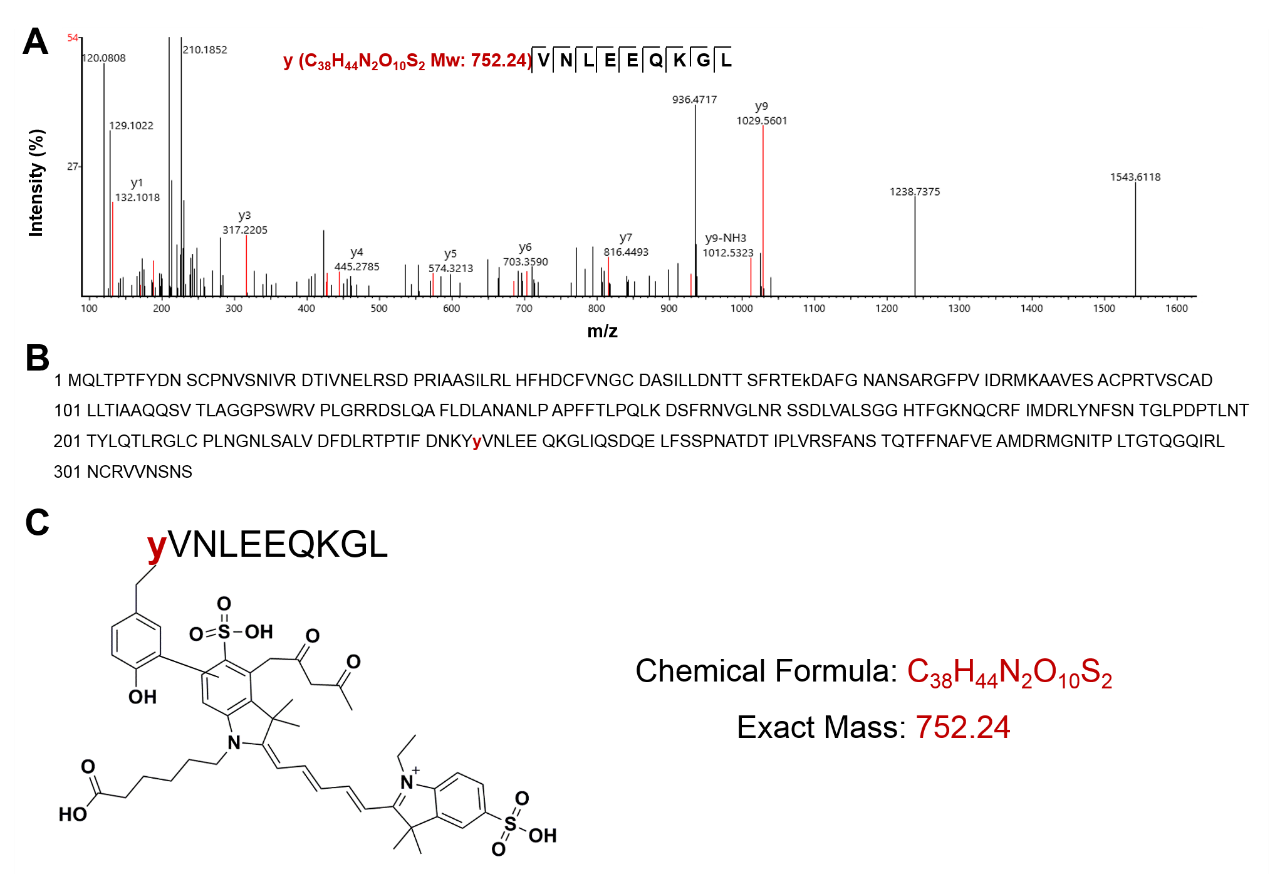
**

**Figure S11.** The peptide modification resolving of the purified HRP products after RIFD reaction with Cy5 and ACAC. (A) MS data analysis. (B) The amino acid sequence of HRP and the Cy5-ACAC co-deposited position. (C) The situation of Cy5-ACAC co-deposited on Y.


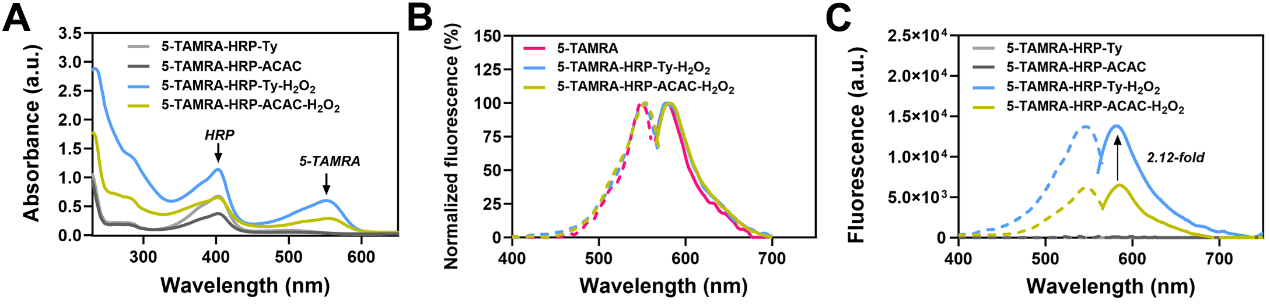


**Figure S12.** (A) UV-vis absorption spectra of purified protein after RIFD with 5-TAMRA under different conditions. (B) Normalized excitation-emission fluorescence spectra of the products compared with pure 5-TAMRA. (C) Fluorescence spectra of the RIFD products.

**
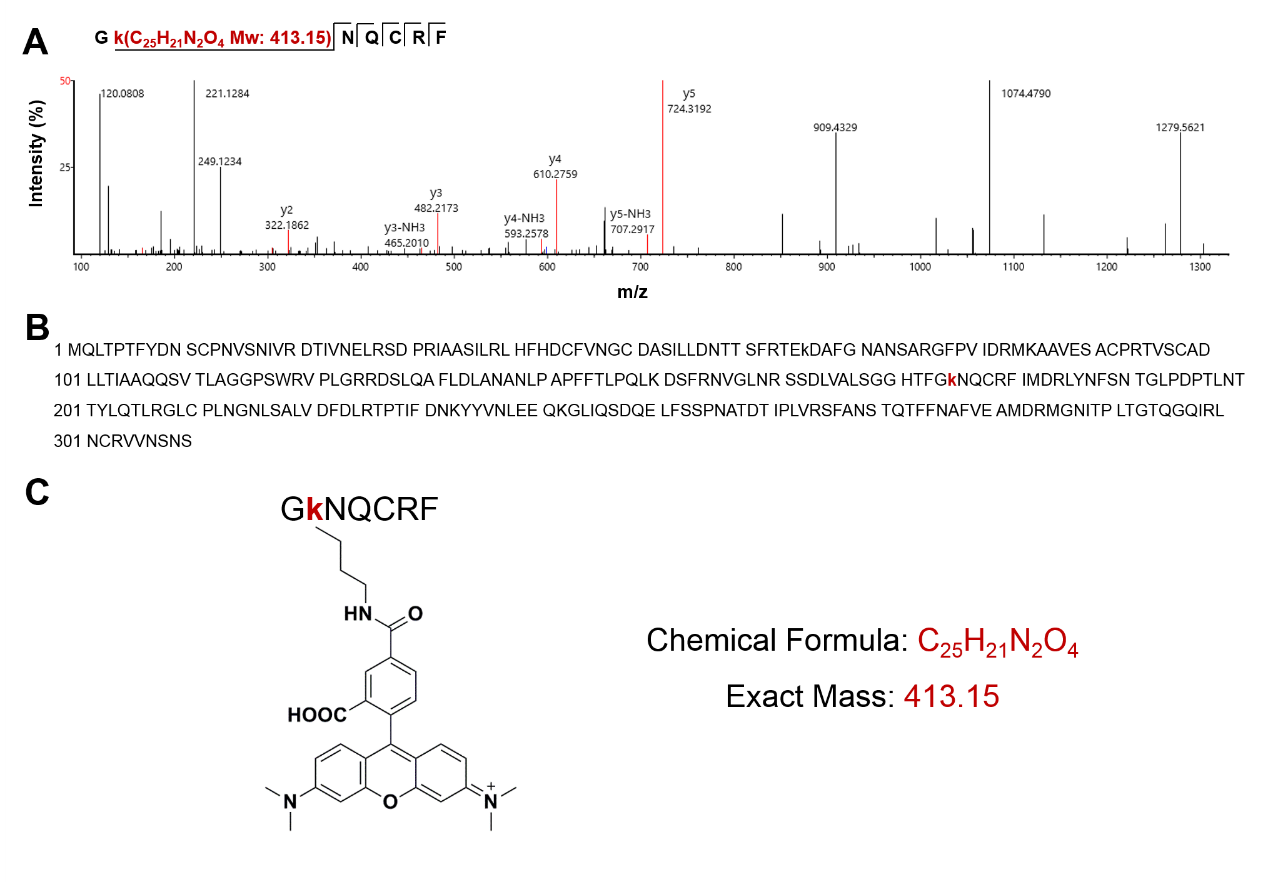
**

**Figure S13.** The peptide modification resolving of the purified HRP products after RIFD reaction with 5-TAMRA and Ty. (A) MS data analysis. (B) The amino acid sequence of HRP and the dye labeled position. (C) The situation of 5-TAMRA labeled on K.

**
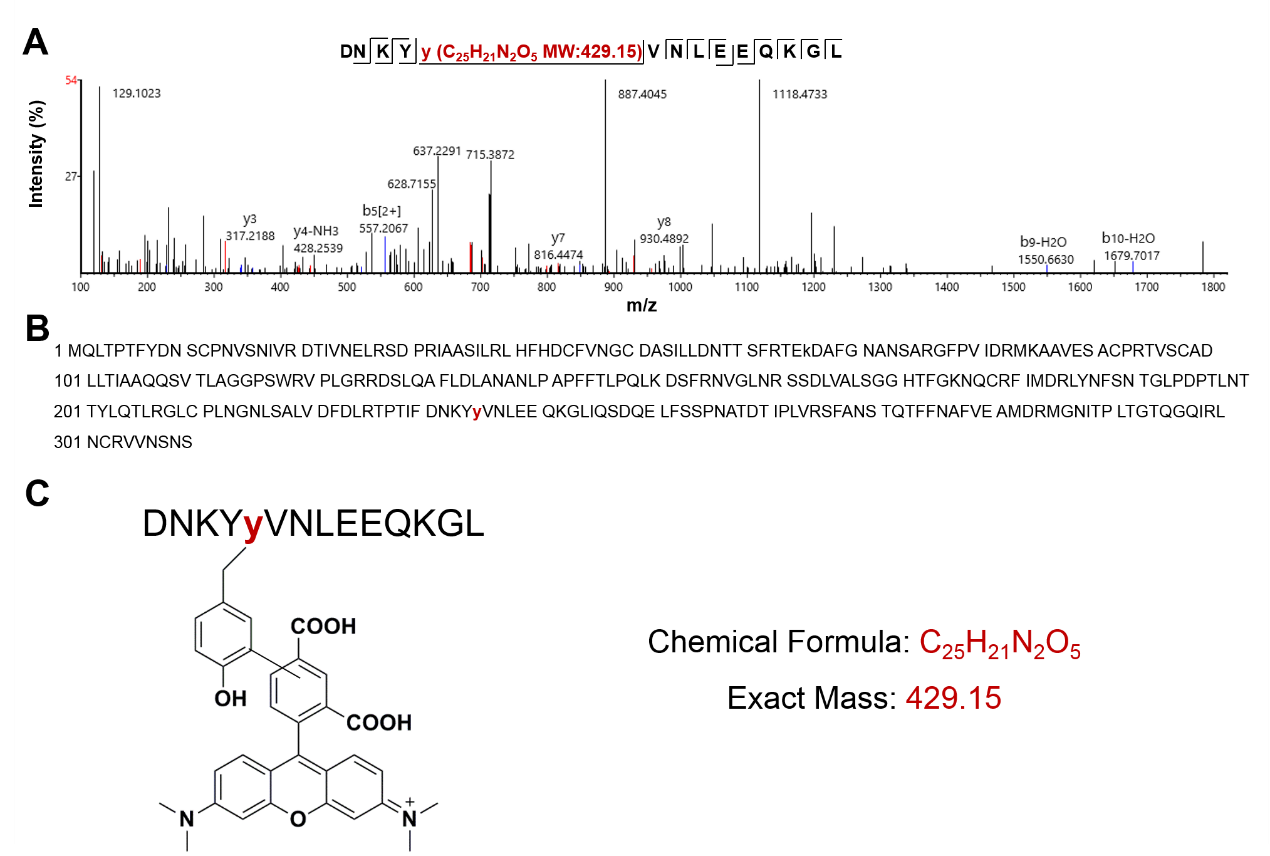
**

**Figure S14.** The peptide modification resolving of the purified HRP products after RIFD reaction with 5-TAMRA and Ty. (A) MS data analysis. (B) The amino acid sequence of HRP and the dye labeled position. (C) The situation of 5-TAMRA labeled on Y.

**
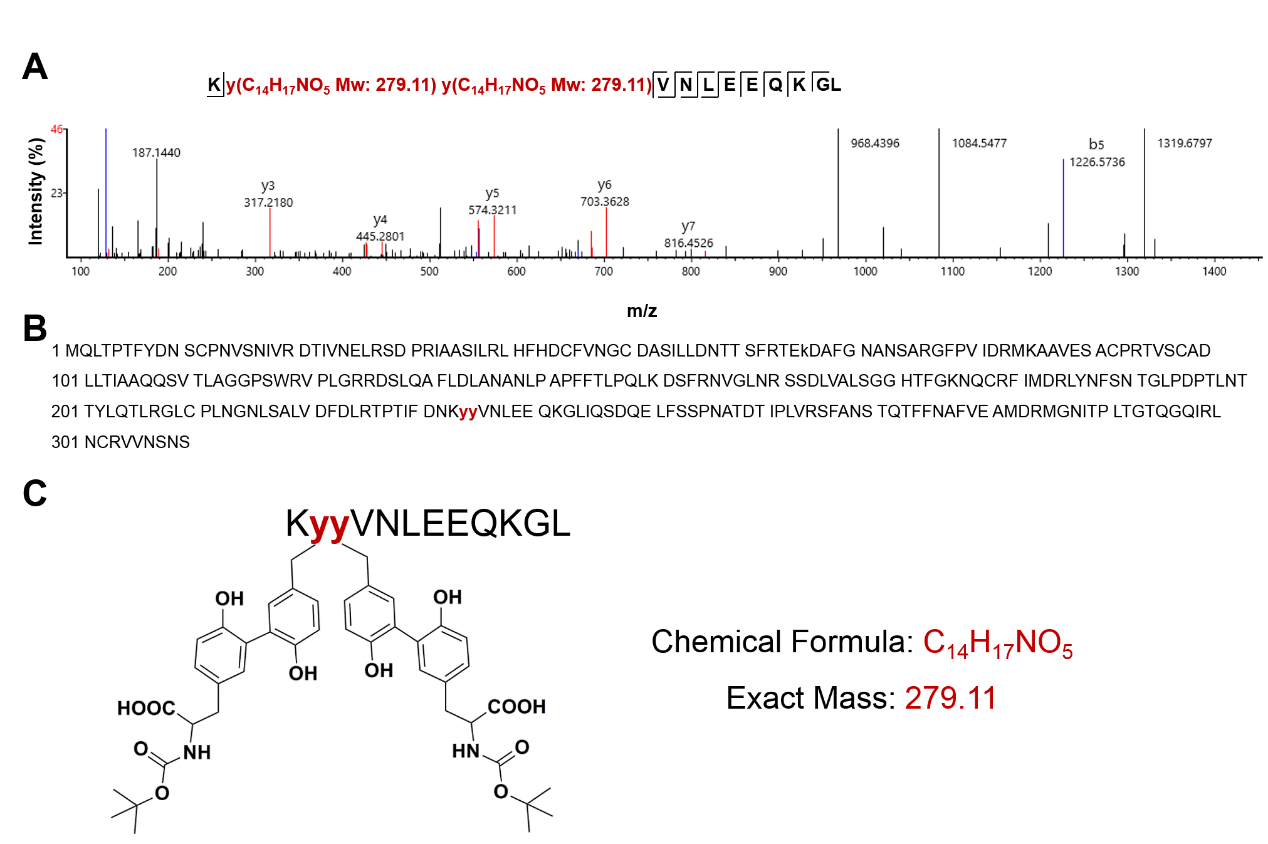
**

**Figure S15.** The peptide modification resolving of the purified HRP products after RIFD reaction with 5-TAMRA and Ty. (A) MS data analysis. (B) The amino acid sequence of HRP and the Ty deposited position. (C) The situation of Ty deposited on Y.

**
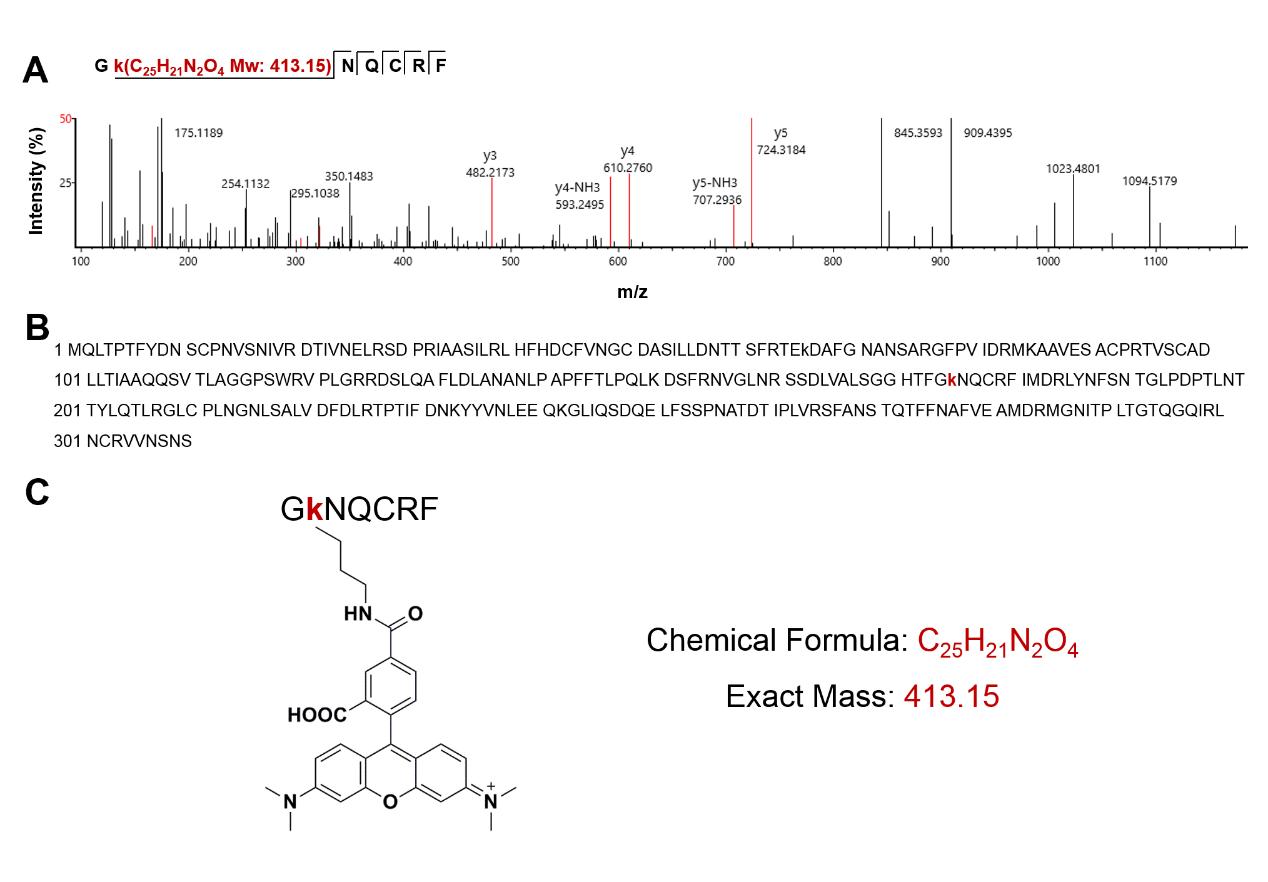
**

**Figure S16.** The peptide modification resolving of the purified HRP products after RIFD reaction with 5-TAMRA and ACAC. (A) MS data analysis. (B) The amino acid sequence of HRP and the dye labeled position. (C) The situation of 5-TAMRA labeled on K.

**
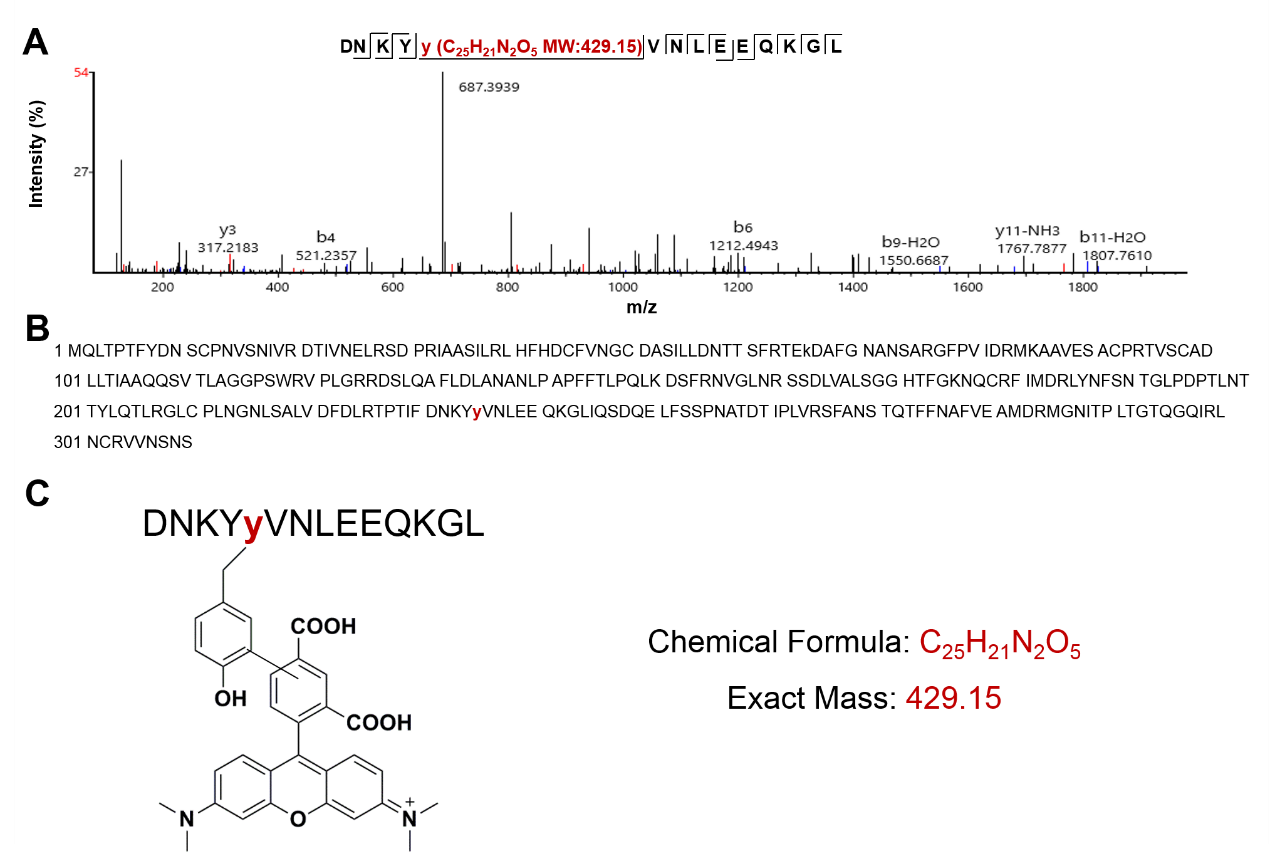
**

**Figure S17.** The peptide modification resolving of the purified HRP products after RIFD reaction with 5-TAMRA and ACAC. (A) MS data analysis. (B) The amino acid sequence of HRP and the dye labeled position. (C) The situation of 5-TAMRA labeled on Y.


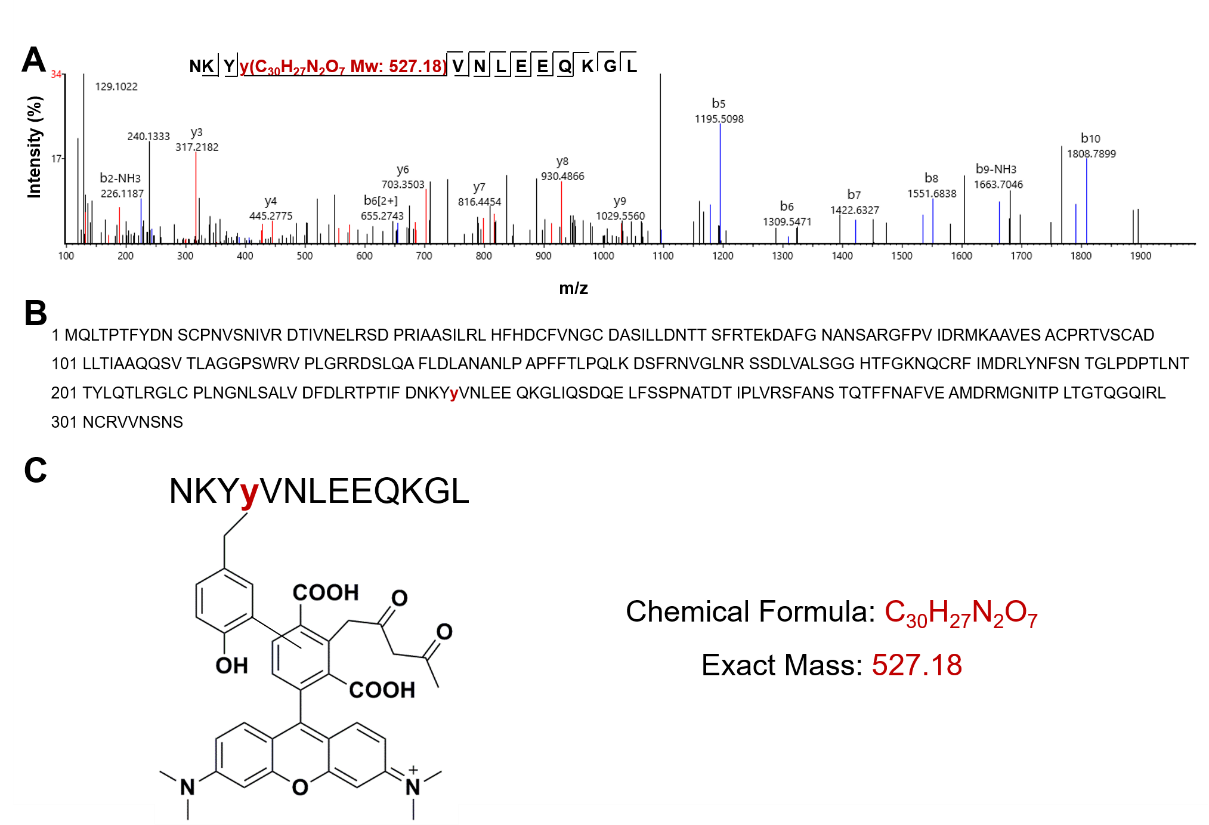


**Figure S18.** The peptide modification resolving of the purified HRP products after RIFD reaction with 5-TAMRA and ACAC. (A) MS data analysis. (B) The amino acid sequence of HRP and the 5-TAMRA-ACAC co-deposited position. (C) The situation of 5-TAMRA-ACAC co-deposited on Y.

**Table S1.** Comparison between classical TSA and the RIFD reaction reported in this work.

| Aspect | Type of reaction | |
| --- | --- | --- |
|  | TSA^[S7-S9]^ | RIFD (this work) |
| Reaction components | Beads, enzyme, H_2_O_2_, Ty-dye / Ty-biotin & SA-dye | Beads, free radicals, dyes |
| Deposition mechanism | Covalent coupling of tyramide with tyrosine | amide condensation (lysine), biphenyl formation (tyrosine), radical-dye co-deposition (tyrosine) |
| Radical substrate | Ty | Ty, ACAC, TPO, etc. |
| Dye format | Pre-conjugated dyes with Ty or SA | Free dye molecules |
| Protein requirement on bead surface | Essential | Enhances but not essential |
| Multiplex compatibility | Yes | Yes |


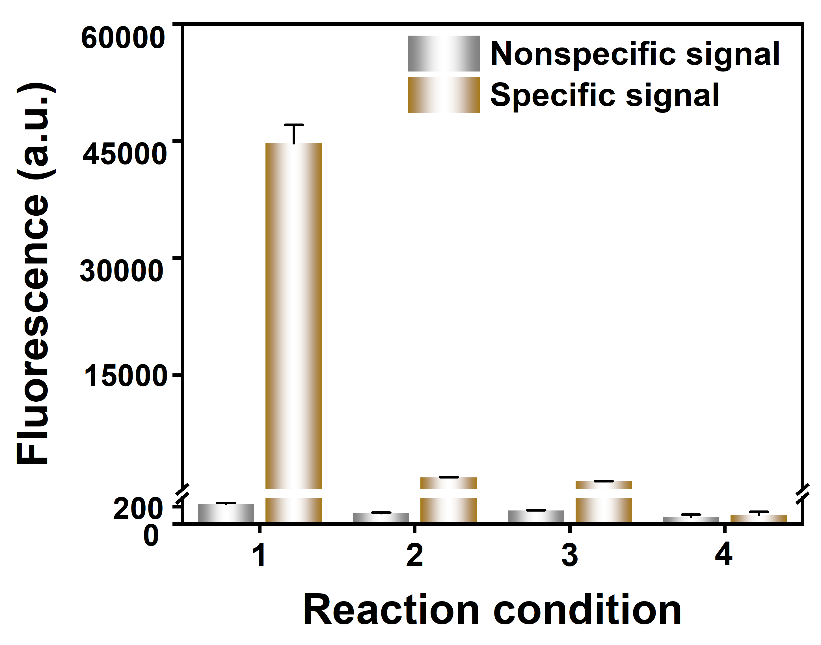


**Figure S19.** FCM analysis of RIFD-based IL-10 immunoassay under different reaction conditions. Mode 1: a mixture of Ty, H_2_O_2_ and Cy5 was normally added for RIFD of 15 min. Mode 2: a mixture of Ty and H_2_O_2_ was first added for 15 min, then Cy5 was supplemented for another 15 min incubation. Mode 3: a mixture of Ty and H_2_O_2_ was first added for 15 min, then the supernatant was removed and Cy5 was added for a further 15 min incubation. Mode 4: a mixture of H_2_O_2_ and Cy5 (without Ty) was added for RIFD of 15 min.


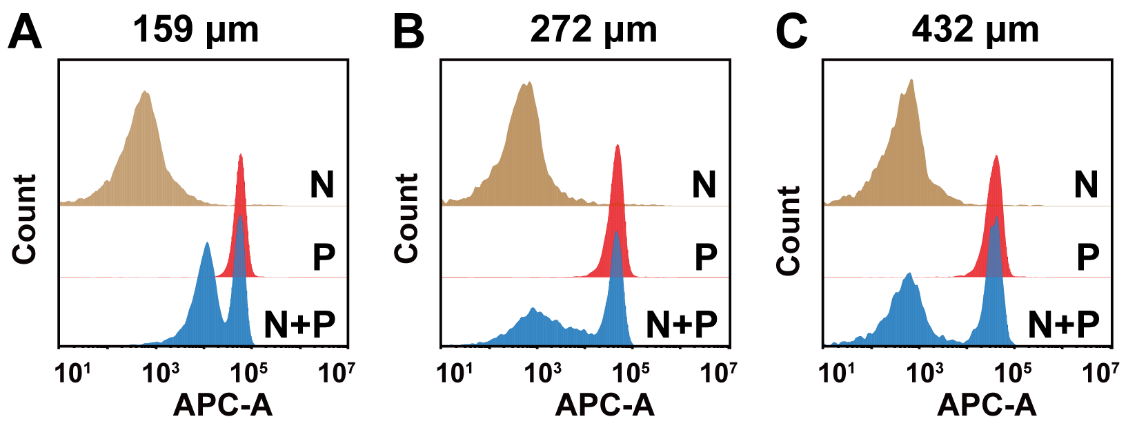


**Figure S20.** FCM analysis of RIFD signals for N, P and N+P mixture with different average distances between beads: (A) 159 μm (2.5×10^5^ beads/mL), (B) 272 μm (5×10^4^ beads/mL), (C) 432 μm (1.25×10^4^ beads/mL).


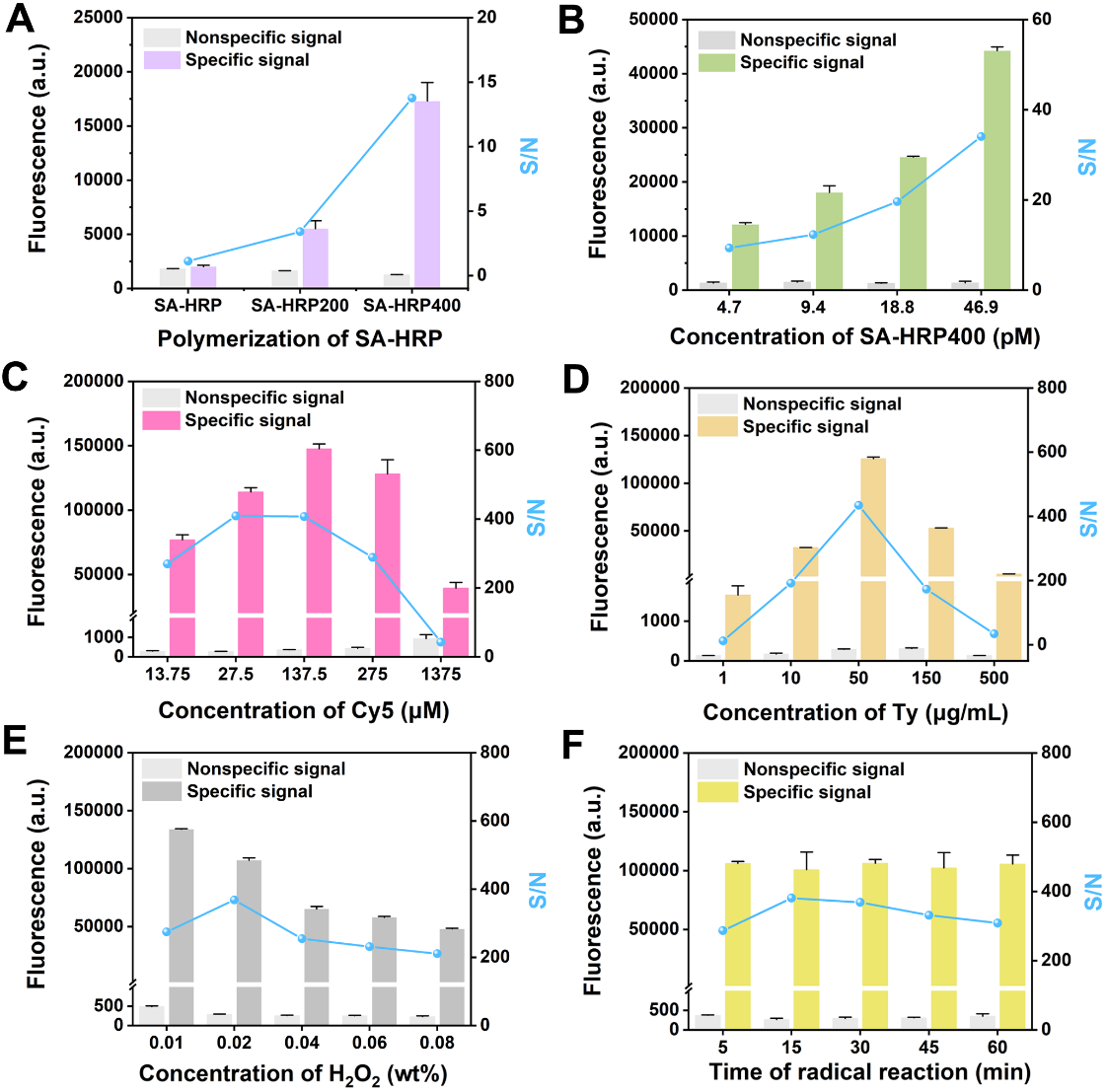


**Figure S21.** Optimization of critical factors for RIFD-based IL-10 immunoassay. (A) Different polymerization of SA-HRP. (B-E) Concentrations of (B) SA-HRP400, (C) Cy5, (D) Ty, and (E) H_2_O_2_. (F) RIFD reaction time. Fluorescence intensities and corresponding S/N ratios are shown.


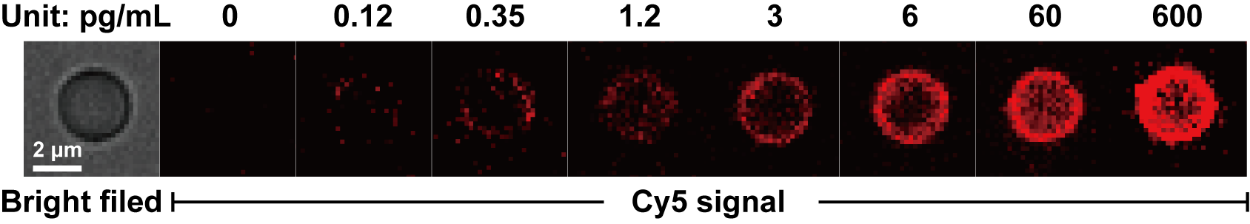


**Figure S22.** LSCM images of beads taken in bright field and Cy5 fluorescence after RIFD-based immunoassay at various IL-10 concentrations (0-600 pg/mL).


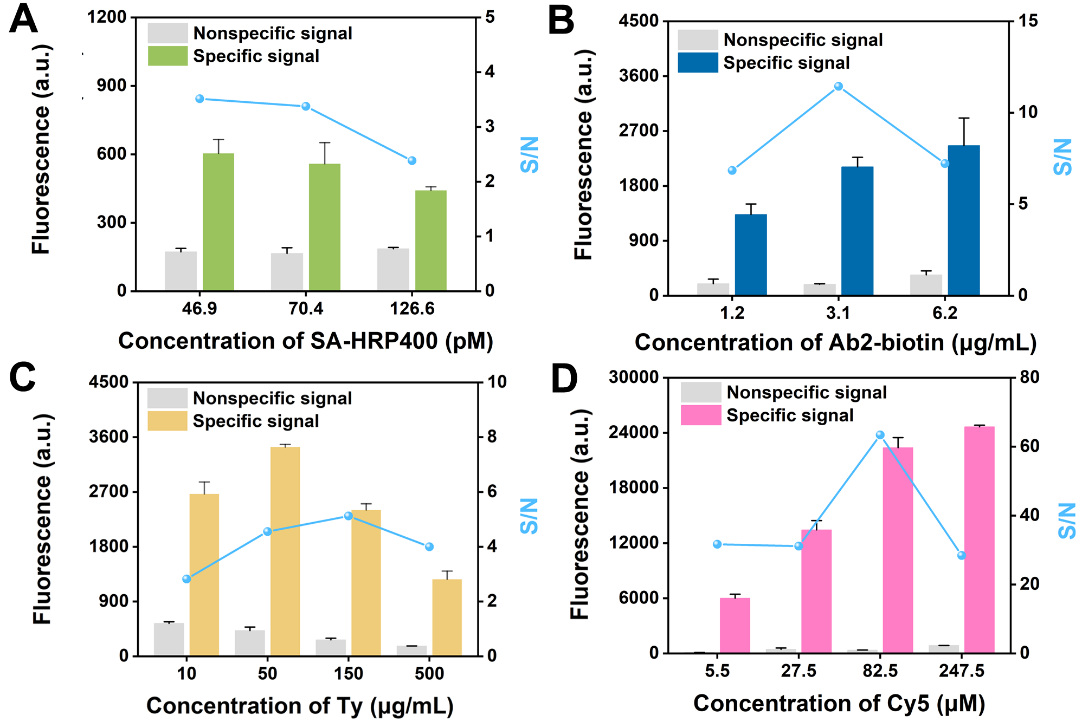


**Figure S23.** Optimization of (A) SA-HRP400, (B) Ab2-biotin, (C) Ty, and (D) Cy5 concentrations for RIFD-based p-Tau217 immunoassay.


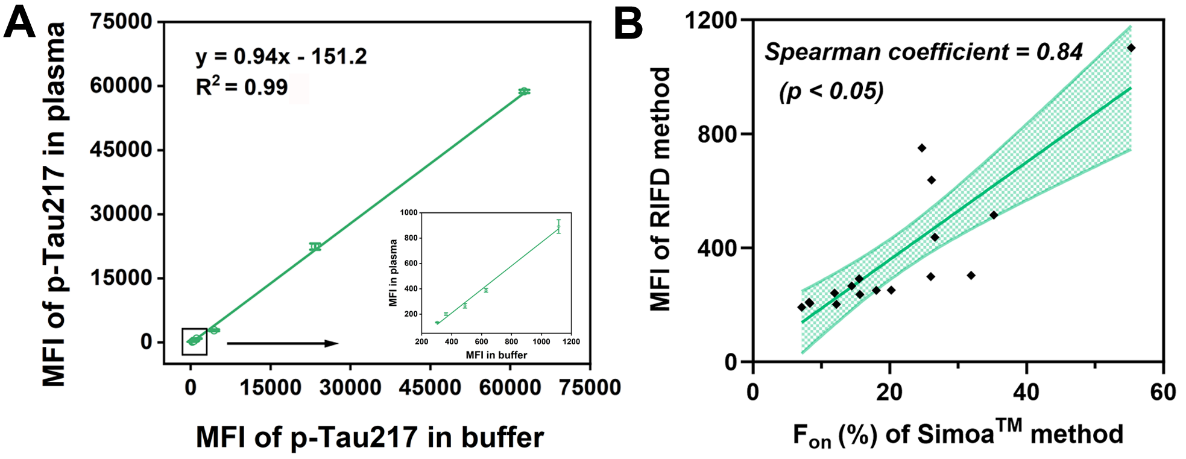


**Figure S24.** (A) Comparison of p-Tau217 detection in plasma and buffer. For the matrix effect evaluation, 23% of plasma in volume was added during the procedure of immunocomplexes formation. (B) Correlation between RIFD-based immunoassay and commercial Simoa^TM^ method for clinical samples of cognitive impairment (n=17). MFI: mean fluorescence intensity. Fon (%): the ratio of positive events on Simoa^TM^.


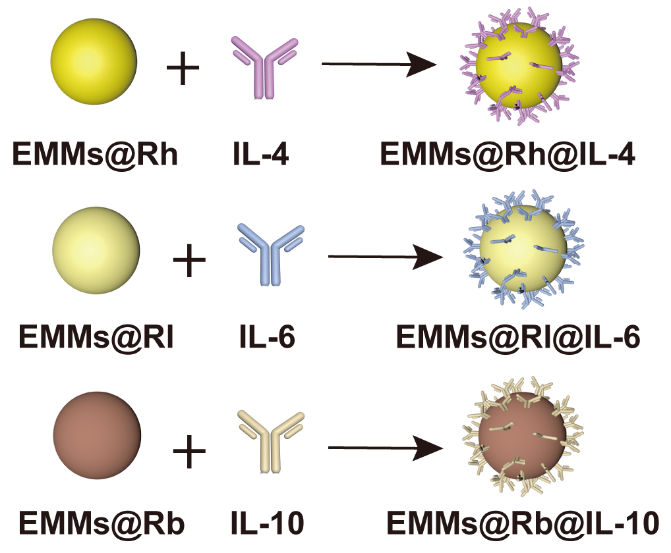


**Figure S25.** Illustration of the corresponding combinations between EMMs and capture antibodies to form barcode beads for different detection indexes.


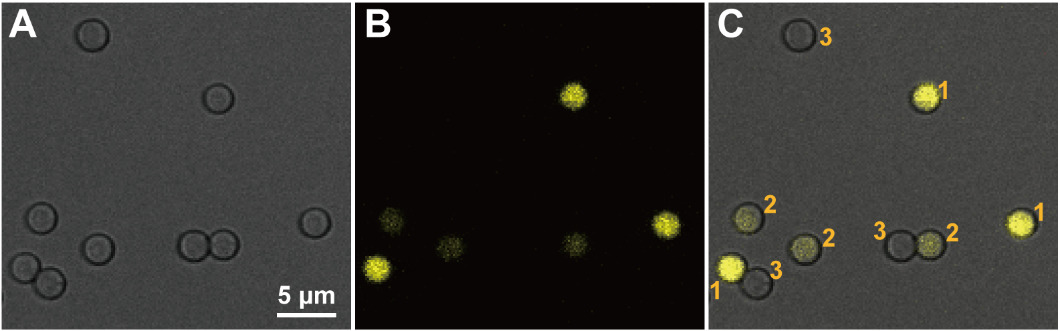


**Figure S26.** LSCM images of mixed barcode beads: (A) bright field, (B) RITC fluorescence, and (C) merged view. The marked numbers represent different barcode beads, 1: EMMs@Rh@IL-4, 2: EMMs@Rl@IL-6, 3: EMMs@Rb@IL-10.

**References**

S1. F. Würthner, T. E. Kaiser, and C. R. Saha-Möller, “J-Aggregates: From Serendipitous Discovery to Supramolecular Engineering of Functional Dye Materials,” Angewandte Chemie International Edition 50 (2011): 3376-3410, <https://doi.org/10.1002/anie.201002307>.

S2. H. Masoomi, Y. Wang, X. X. Fang, et al., “Ultrabright Dye-Loaded Spherical Polyelectrolyte Brushes and Their Fundamental Structure-Fluorescence Tuning Principles,” Nanoscale 11 (2019): 14050-14059, <https://doi.org/10.1039/C9NR02168J>.

S3. E. J. M. Speel, A. H. N. Hopman, and P. Komminoth, “Amplification Methods to Increase the Sensitivity of in Situ Hybridization: Play CARD(S),” Journal of Histochemistry and Cytochemistry 47 (1999): 281-288, <https://doi.org/10.1177/002215549904700302>.

S4. H. Zhang, L. Liu, X. Fu, and Z. J. Zhu, “Microfluidic Beads-Based Immunosensor for Sensitive Detection of Cancer Biomarker Proteins Using Multienzyme-Nanoparticle Amplification and Quantum Dots Labels,” Biosensors and Bioelectronics 42 (2013): 23-30, <https://doi.org/10.1016/j.bios.2012.11.010>.

S5. J. Y. Zhang, J. C. Wu, C. Chen, et al., “A Micro-Chamber Free Digital Bio-Detection for Multiplexed and Ultrasensitive Immunoassay Based on Encoded Magnetic Microbeads and Tyramide Signal Amplification Strategy,” Talanta 262 (2023): 124685,

<https://doi.org/10.1016/j.talanta.2023.124685>.

S6. H. B. Dunford and J. S. Stillman, “Function and Mechanism of Action of Peroxidases,” Coordination Chemistry Reviews 19 (1976): 187-251,

<https://doi.org/10.1016/S0010-8545(00)80316-1>.

S7. W. J. Xue, L. Wang, K. X. Yi, L. Y. Sun, H. Z. Ren, and F. K. Bian, “Hepatocellular Carcinoma Biomarkers Screening Based on Hydrogel Photonic Barcodes with Tyramine Deposition Amplified ELISA,” Biosensors and Bioelectronics 255 (2024): 116270, <https://doi.org/10.1016/j.bios.2024.116270>.

S8. D. S. Chen, X. B. Zhang, L. P. Zhu, C. H. Liu, and Z. P. Li, “All on Size-Coded Single Bead Set: A Modular Enrich-Amplify-Amplify Strategy for Attomolar Level Multi-Immunoassay,” Chemical Science 13 (2022): 3501-3506,

<https://doi.org/10.1039/D1SC07048G>.

S9. G. P. Anderson and C. R. Taitt, “Amplification of Microsphere-Based Microarrays Using Catalyzed Reporter Deposition,” Biosensors and Bioelectronics 24 (2008): 324-328, <https://doi.org/10.1016/j.bios.2008.03.045>.
